# Gadoxetic Acid–enhanced MRI Radiomics Features of Tumor Margins for Predicting High-Risk Solitary Hepatocellular Carcinoma Aggressiveness and Prognosis

**DOI:** 10.1148/rycan.250220

**Published:** 2026-01-23

**Authors:** Can Yu, Xinxin Wang, Shuli Tang, Yan Li, Shuai Han, Qiuju Zhang, Jinrong Qu, Haitao Xu, Yang Zhou

**Affiliations:** ^1^Department of Radiology, Harbin Medical University Cancer Hospital, Harbin, China; ^2^Department of Outpatient Chemotherapy, Harbin Medical University Cancer Hospital, Harbin, China; ^3^Department of Hepatobiliary and Pancreatic Surgery, Harbin Medical University Cancer Hospital, Harbin, China; ^4^Health Management Centre, Harbin Medical University Cancer Hospital, Harbin, China; ^5^Department of Biostatistics, Public Health School of Harbin Medical University, Harbin, China; ^6^Department of Radiology, The Affiliated Cancer Hospital of Zhengzhou University & Henan Cancer Hospital, Zhengzhou, China

**Keywords:** MRI, Machine Learning, Radiomics, Radiogenomics, Abdomen/GI, Liver, Surgery, High-Risk Solitary Hepatocellular Carcinoma, Tumor Margin, Microvascular Invasion, Gd-EOB-DTPA-enhanced MRI, OATP1B3

## Abstract

**Purpose:**

To develop a radiomics model based on hepatobiliary phase gadolinium ethoxybenzyl-diethylenetriaminepentaacetic acid (EOB)–enhanced MRI features at the tumor margin to predict microvascular invasion in high-risk solitary hepatocellular carcinoma (HR-sHCC), determine the optimal margin region, and explore the underlying biologic mechanisms.

**Materials and Methods:**

This retrospective study included patients with HR-sHCC from three medical centers between April 2015 and December 2022. Radiomics features were extracted from 121 volumes of interest (VOIs) at the tumor margin at EOB MRI. Nine combinations of statistical and machine learning methods were used to construct and validate the optimal margin region–based radiomics model. Model performance was assessed using the area under the receiver operating characteristic curve (AUC), and patient stratification was evaluated with Kaplan–Meier and log-rank analyses. RNA sequencing data underwent differential expression analysis with DESeq2, followed by Kyoto Encyclopedia of Genes and Genomes (ie, KEGG) and Gene Ontology (ie, GO) enrichment, and immune cell infiltration was assessed using xCell and EPIC.

**Results:**

A total of 436 patients (mean age, 57.7 years ± 8.8 [SD]; 352 male) were included: 254 in the training, 108 in the internal test, and 74 in the external test cohorts. Receiver operating characteristic analysis showed AUCs of 0.80 (95% CI: 0.74, 0.86), 0.76 (95% CI: 0.66, 0.85), and 0.72 (95% CI: 0.58, 0.86), respectively. The model effectively stratified patients by overall and disease-free survival (all *P* < .05). RNA sequencing revealed extracellular matrix remodeling, transforming growth factor–β signaling, and M2 macrophage infiltration in high optimal margin region–score tumors.

**Conclusion:**

The optimal margin region–based radiomics model, derived from EOB MRI, effectively captured tumor margin heterogeneity.

**Keywords:** MRI, Machine Learning, Radiomics, Radiogenomics, Abdomen/GI, Liver, Surgery, High-Risk Solitary Hepatocellular Carcinoma, Tumor Margin, Microvascular Invasion, Gd-EOB-DTPA-enhanced MRI, OATP1B3

© The Author(s) 2026. Published by the Radiological Society of North America under a CC BY 4.0 license.

*Supplemental material is available for this article.*

## Graphical abstract

**Figure:**
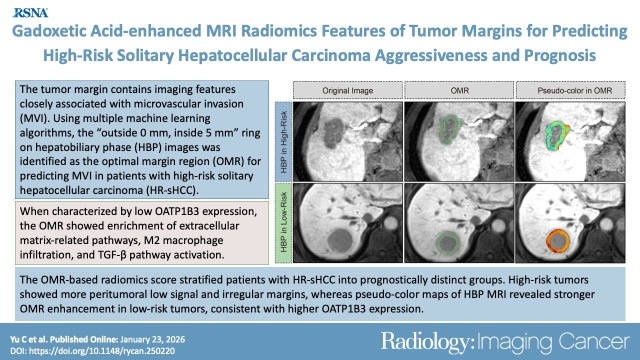

**Summary**

A gadoxetic acid–enhanced MRI–based radiomics model captured tumor margin heterogeneity to predict high-risk solitary hepatocellular carcinoma microvascular invasion and prognosis, with strong performance across cohorts and biologic support from RNA sequencing and histopathologic analyses.

**Key Points**

■ A novel tumor margin region (outside 0 mm, inside 5 mm on hepatobiliary phase images) was identified as the optimal margin region for predicting microvascular invasion in high-risk solitary hepatocellular carcinoma (HR-sHCC), achieving area under the receiver operating characteristic curves of 0.80, 0.76, and 0.72 in the training, internal test, and external test cohorts, respectively.
■ An optimal margin region–based radiomics score effectively stratified patients with HR-sHCC into high- and low-risk groups with significantly different overall survival and disease-free survival (all *P* < .05); the high-risk group showed more peritumoral low signal (45 of 124 [36%] vs 50 of 238 [21%]) and irregular morphology (50 of 124 [40%] vs 136 of 238 [57%]).
■ Biologic analysis showed that low OATP1B3 expression (*P* < .05) and extracellular matrix enrichment (*P* < .05) in the optimal margin region were linked to tumor invasiveness, accompanied by increased M2 macrophage infiltration (*P* = .012) and transforming growth factor–β pathway activation.

## Introduction

Hepatocellular carcinoma (HCC) is one of the most prevalent and deadly malignancies worldwide (1). According to the IMbrave050 trial, high-risk recurrence is defined by either a solitary tumor > 2 cm in size (high-risk solitary HCC [HR-sHCC]) or the presence of multiple tumors (2). For patients with HR-sHCC, surgery is often the preferred treatment. However, because of the high heterogeneity of tumors, their biologic behavior and aggressiveness vary substantially, directly impacting disease prognosis (3). Microvascular invasion (MVI) is a key hallmark of the aggressive biologic behavior of HCC, with its occurrence influenced by multiple factors, such as the high proliferative activity of tumor cells and an immunosuppressive tumor microenvironment (4,5). These factors collectively increase the invasiveness of HCC, accelerate local recurrence, and substantially increase the risk of postoperative distant metastasis, ultimately affecting patient outcomes (6). Therefore, for patients with HR-sHCC, accurately assessing tumor heterogeneity is crucial for risk stratification and the optimization of personalized treatment strategies, which are essential for improving long-term survival rates.

The tumor margin region represents the transition zone where normal cells transform into cancer cells, serving as the most actively invasive part of the tumor (7). Previous studies have shown that the characteristics of the tumor margin, such as immune cell infiltration, immune barriers, and tissue stiffness, are closely associated with differences in the biologic behavior of HCC. However, detecting these features often requires invasive procedures, such as surgery or biopsy (8–10). Consequently, noninvasive and accurate methods to evaluate these tumor margin characteristics have become a critical area of research.

In China, radiologic examinations for tumors are nearly universally available, and these examinations not only are noninvasive but also show great potential in predicting tumor aggressiveness and disease prognosis (11,12). Imaging features of HCC at the tumor margins, such as the halo sign, incomplete capsule, and radiomics features, have been shown to be closely associated with tumor aggressiveness and prognosis (13,14). However, these imaging findings have yet to make a substantial impact in clinical practice because of a lack of clear biologic explanations.

Compared with conventional gadolinium-based contrast agents, gadoxetic acid (gadolinium ethoxybenzyl-diethylenetriaminepentaacetic acid, or Gd-EOB-DTPA) is a molecularly targeted contrast agent that specifically binds to organic anion transporting polypeptide (OATP) receptors on the surface of hepatocytes (15,16). This binding suggests that Gd-EOB-DTPA (EOB)–enhanced MRI could not only improve lesion detection and boundary delineation but also enable simultaneous assessment of liver function and provide direct insights into underlying molecular mechanisms, thereby offering new opportunities for diagnosis and therapy.

We aimed to develop a novel MVI prediction model based on the radiomics features of tumor margins of HR-sHCC at EOB MRI, identify the optimal margin region for MVI prediction, and elucidate the biologic mechanisms underlying these features.

## Materials and Methods

### Study Cohorts

Our retrospective study involved consecutive patients with HCC who underwent EOB MRI scans 20 minutes after contrast agent injection, before surgery, at three medical centers in China between April 2015 and December 2022. Inclusion criteria were: *(a)* EOB MRI of the liver conducted within 2 weeks before surgery, *(b)* solitary HCC confirmed with histopathology, and *(c)* no treatment before surgery. Exclusion criteria were as follows: *(a)* poor imaging quality, *(b)* tumor diameter less than 2 cm, *(c)* incomplete clinical or pathologic information, *(d)* a history of malignancies, and *(e)* evidence of gross vessel invasion at MRI.

Patients from Harbin Medical University Cancer Hospital were randomly assigned to a training cohort (254 patients) and an internal test cohort (108 patients). An external test cohort comprised 74 patients from Henan Cancer Hospital and Shangdong Cancer Hospital center. Between January 2023 and December 2024, 63 Harbin Medical University Cancer Hospital patients with HR-sHCC, meeting the same criteria, were included in the RNA sequencing cohort; 30 of them formed the immunohistochemistry cohort. Genetic data from 368 patients with HCC in The Cancer Genome Atlas (TCGA) served as an additional test cohort. Multiregion RNA sequencing was performed on 16 HR-sHCC tissue samples for tumor-specific analysis. The study was approved by all centers’ ethics committees; informed consent was waived due to the study’s retrospective design. All radiomics, optimal margin region selection, and RNA sequencing/immunohistochemistry analyses are novel.

### Clinical Outcome and Follow-up

The primary study end point was the diagnostic performance of the EOB MRI–based radiomics model for predicting MVI, with histopathologic assessment serving as the reference standard. The secondary end points were overall survival and disease-free survival, evaluated using Kaplan–Meier and log-rank analyses. In the absence of recurrence, the date of death or the last follow-up was considered the study end point. Routine follow-ups were conducted every 3 to 6 months after treatment until the patient’s death. For patients lost to follow-up, the final follow-up time was defined as the last time point at which they were known to be under observation.

### MRI Acquisition and Segmentation

We collected hepatobiliary phase MRI sequences from examinations performed within 2 weeks before surgery using EOB (Primovist/Eovist; Bayer) as the contrast agent. MRI examination details for all centers are provided in Table S1. We used 3D Slicer software (version 5.6.1; *www.slicer.org*, Brigham and Women’s Hospital) to segment the lesions and define their margins, creating the original lesion volumes of interest (VOIs). We then modified the tumor margins using the SimpleITK package in Python (version 3.8.19; Python Software Foundation), with inward erosion and outward expansion applied within a range of 0–10 mm, resulting in a total of 120 margin regions (as illustrated in Fig S1) (17). Combined with the original lesion VOI, each patient’s lesion included 121 VOIs. We resampled all images using the SciPy package. Two experienced radiation oncologists (C.Y., with 6 years of experience, and X.W., with 8 years of experience) independently delineated the original lesion VOIs. Their delineations were individually reviewed and verified by a senior radiation oncologist (Y.Z., >15 years of experience) to assess intra- and interreader consistency. We quantitatively evaluated the consistency of the original lesion VOI segmentation by determining the intraclass correlation coefficient, ensuring high reproducibility and accuracy of the segmentation process.

### Radiomics Feature Engineering

We used the pyradiomics package in Python to extract radiomics features from the 121 regions for each patient. We calculated all features, except for shape features, from both the original and filtered images. In total, we extracted 2060 radiomics features from each VOI. From each VOI, we retained only features with an intraclass correlation coefficient greater than 0.75 for subsequent analysis.

### Machine Learning Signature Construction

We used nine combinations of statistical and machine learning methods for feature selection: *(a)* correlation + least absolute shrinkage and selection operator (LASSO); *(b)* logistic regression + LASSO; *(c)* correlation + LASSO + stepwise; *(d)* logistic regression + LASSO + stepwise; *(e)* extreme gradient boosting (ie, XGBoost) + LASSO + stepwise; *(f)* random forest + LASSO + stepwise; *(g)* gradient boosting machine + LASSO + stepwise; *(h)* support vector machine + LASSO + stepwise; and *(i)* LASSO + stepwise. The specific definitions of each method are detailed in Table S2. We generated various radiomics feature combinations for the training cohort, performing 10-fold cross-validation to evaluate each method, and we calculated area under the receiver operating characteristic curve (AUC) values for each region on the basis of these feature sets. We determined the optimal method combination and optimal margin region by ranking the AUC values from the internal test cohort, and we validated the model for the best margin region in the internal test cohort. We completed feature selection, model construction, and optimal margin region identification within the training cohort. We used a logistic regression approach for modeling that was based on the selected features. We divided patients into optimal margin region high-risk and low-risk groups on the basis of the optimal cutoff value derived from the maximum Youden index in the training cohort.

### Exploring Biologic Functions

HR-sHCC tissue samples were taken from 63 patients who underwent liver resection at Harbin Medical University Cancer Hospital between January 2023 and December 2024, and we performed RNA sequencing on these samples. We grouped patients in the RNA sequencing cohort on the basis of the optimal cutoff value determined in the training cohort into optimal margin region high-risk and low-risk groups. We performed differential gene expression analysis using the DESeq2 package (genes with |log_2_ fold change| > 1 and *P* < .05 were considered differentially expressed), followed by pathway enrichment analysis via the Kyoto Encyclopedia of Genes and Genomes (KEGG) and gene-level functional enrichment analysis via Gene Ontology, as well as immune cell infiltration analysis using xCell *(https://comphealth.ucsf.edu/app/xcell)* to explore the biologic functions of these groups (18–20). The biologic function results were validated with data from 368 patients with HCC in TCGA. Multiregion RNA sequencing in 16 patients with HR-sHCC captured spatial heterogeneity. Tumor microenvironment scores (ImmuneScore, StromaScore, MicroenvironmentScore) were calculated using xCell; single-sample Gene Set Enrichment Analysis (ie, ssGSEA) assessed KEGG pathway enrichment; EPIC estimated tumor microenvironment–infiltrating cell composition (21–23).

### Laboratory Immunohistochemistry and Staining

We obtained HR-sHCC tissue specimens from 30 patients selected from the RNA sequencing cohort. We confirmed the enrichment results through immunohistochemistry and tissue staining (24). We performed immunohistochemistry staining for OATP1B3 and Masson trichrome (catalog no. G1346; Solarbio) and PicroSirius red staining (catalog no. BL1194B; Biosharp) on tissue slices from tumor margins obtained after surgery (25,26). We performed immunohistochemistry staining for OATP1B3 (catalog no. 66381-1-Ig; Proteintech) on the paraffin sections according to the manufacturer’s instructions. To determine the percentage of stained areas of regions, we used the color segmentation function in ImageJ software (version 1.54; National Institutes of Health) to identify and quantify the staining of different structures. We compared the percentages of stained areas of regions across different groups, and then we performed distribution analysis and intergroup comparison of these percentages.

### Statistical Analysis

Continuous variables are presented as means ± SDs, while categorical variables are presented as frequencies and percentages. We conducted survival and recurrence risk analyses using the Kaplan–Meier method combined with the log-rank test. We computed and compared the AUCs of radiomics models derived from distinct tumor margin regions in both the training and internal test cohorts, and 95% CIs were calculated using the ci.auc function. We used SHapley Additive exPlanation to display the contribution of each radiomics feature (27). We calculated *P* values using the χ^2^ test or Fisher exact test, as appropriate, to compare categorical variables between the optimal margin region low-risk and high-risk groups. We considered a *P* value of less than .05 statistically significant. We performed image segmentation and radiomics feature extraction using Python (version 3.8.19), and we completed all statistical analyses and graph generation using R software (version 4.3.1) and GraphPad Prism (version 8.0.2; GraphPad Software). The training cohort included 254 patients, and the internal test cohort included 108 patients, randomly assigned in a 7:3 ratio. This sample size was deemed adequate based on the number of events and comparable prior radiomics studies of MVI prediction, providing sufficient power for model development and evaluation. All analyses were performed by two authors with more than 6 (Y.L.) and 10 (Q.Z.) years of experience in biostatistics.

## Results

### Patient Characteristics

Between April 2015 and December 2022, of 735 consecutive patients with HCC from three centers, 37 were excluded for poor imaging quality, 73 for tumor diameter < 2 cm, 125 for incomplete clinical or pathologic information, 36 for a history of malignancy, and 28 for gross vessel invasion at MRI, leaving 436 patients (mean age, 57.7 years ± 8.8 [SD]; 352 male, 84 female) for analysis. The number of patients with MVI in the training, internal test, and external test cohorts was 76, 39, and 24, respectively. At the last follow-up, there were 57, 27, and 20 deaths in the training, internal test, and external test cohorts, respectively, with a median follow-up time of 27.1 months (IQR, 18.4–49.5 months) for patients who were alive, and tumor recurrence events were observed in 95, 42, and 14 patients, respectively, with a median follow-up time of 24.6 months (IQR, 13.0–46.0 months) for patients without recurrence. The numbers of patients lost to follow-up in the three cohorts were 57, 13, and 16, respectively. Detailed baseline clinical characteristics are presented in Table 1.

**Table 1:** Baseline Patient Characteristics

Characteristic	Training Cohort (*n* = 254)	Internal Test Cohort (*n* = 108)	External Test Cohort (*n* = 74)
Age (y)*	59 (52–64)	58 (53–62)	57 (53–62.75)
Tumor size (mm)*	41 (29–62)	43 (28–64.5)	42 (30.25–61.75)
Sex			
Female	54 (21.3)	23 (21.3)	7 (9.5)
Male	200 (78.7)	85 (78.7)	67 (90.5)
AFP level (ng/mL)^†^			
≤400	187 (73.6)	76 (70.4)	61 (82.4)
>400	67 (26.4)	32 (29.6)	13 (17.6)
Child-Pugh classification			
A	239 (94.1)	99 (91.7)	65 (87.8)
B	15 (5.9)	9 (8.3)	9 (12.2)
Cirrhosis			
No	29 (11.4)	12 (11.1)	8 (10.8)
Yes	225 (88.6)	96 (88.9)	66 (89.2)
Hepatitis			
Hepatitis B	211 (83.1)	97 (89.8)	67 (90.5)
Hepatitis C	16 (6.3)	5 (4.6)	5 (6.8)
None	27 (10.6)	6 (5.6)	2 (2.7)
AST level (U/L)^‡^			
≤40	161 (63.4)	68 (63.0)	55 (74.3)
>40	93 (36.6)	40 (37.0)	19 (25.7)
ALT level (U/L)^‡^			
≤40	157 (61.8)	70 (64.8)	55 (74.3)
>40	97 (38.2)	38 (35.2)	19 (25.7)
GGT level (U/L)^‡^			
≤60	154 (60.6)	63 (58.3)	55 (74.3)
>60	100 (39.4)	45 (41.7)	19 (25.7)
PLT (U/L)			
≤150	122 (48.0)	50 (46.3)	32 (43.2)
>150	132 (52.0)	58 (53.7)	42 (56.8)
MVI			
No	178 (70.1)	69 (63.9)	50 (67.6)
Yes	76 (29.9)	39 (36.1)	24 (32.4)
Histologic grade			
Well and moderately to well	72 (28.3)	17 (15.7)	1 (1.4)
Moderately	126 (49.6)	62 (57.4)	59 (79.7)
Moderately to poorly and poorly	56 (22.0)	29 (26.9)	14 (18.9)

Note.—Unless otherwise noted, data are presented as numbers, with percentages in parentheses. AFP = α-fetoprotein, ALT = alanine aminotransferase, AST = aspartate aminotransferase, GGT = γ-glutamyltransferase, MVI = microvascular invasion, PLT = platelet count.

* Data are presented as medians, with IQRs in parentheses.

^†^ SI conversion factor: To convert nanograms per milliliter to micrograms per liter (μg/L), multiply by 1.0.

^‡^ SI conversion factor: To convert units per liter to microkatal per liter (μkat/L), multiply by 0.0167.

**Figure 1:**
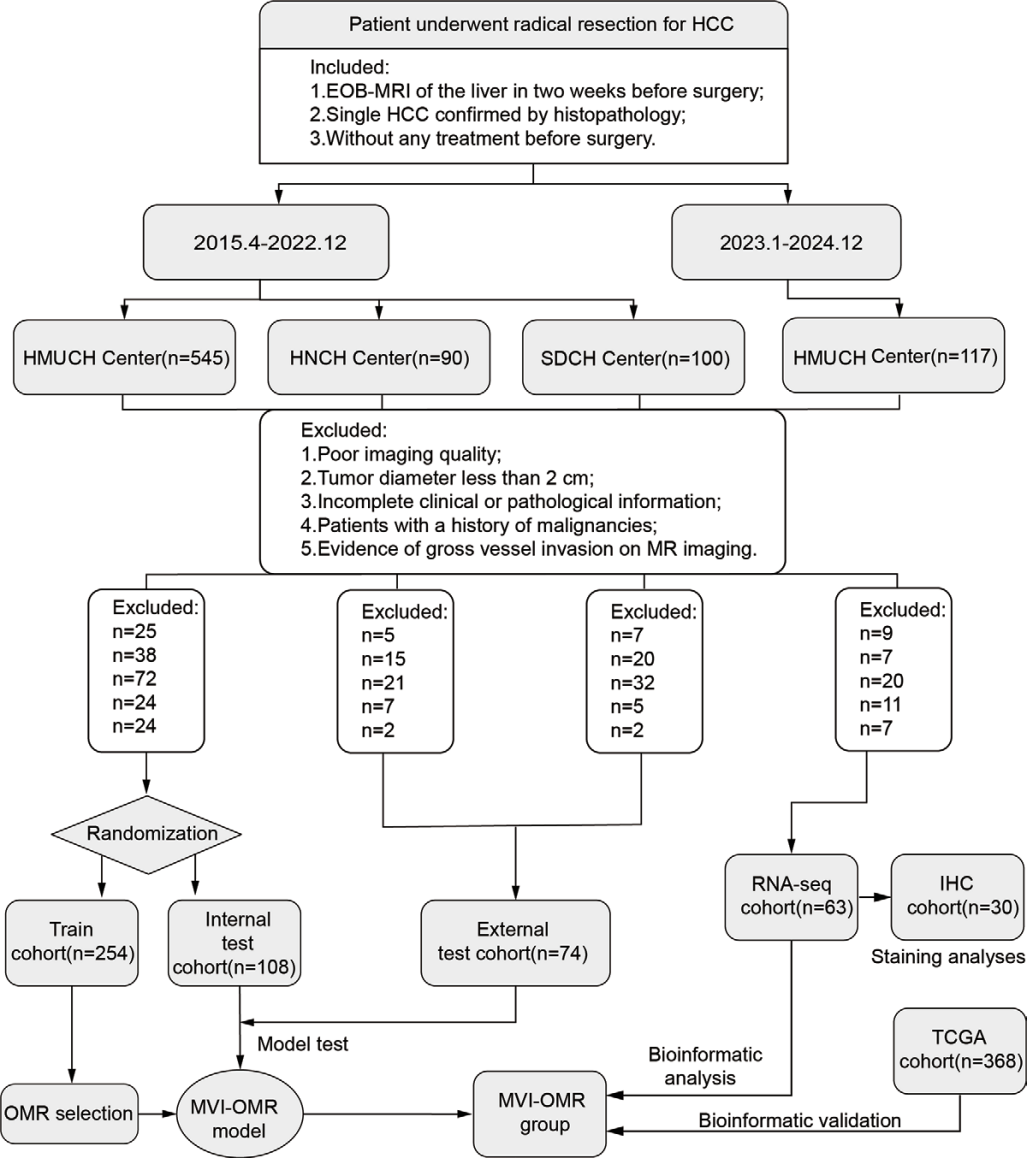
Patient selection and study design. EOB = gadolinium ethoxybenzyl-diethylenetriaminepentaacetic acid–enhanced, HCC = hepatocellular carcinoma, HMUCH = Harbin Medical University Cancer Hospital, HNCH = Henan Cancer Hospital, IHC = immunohistochemistry, MVI = microvascular invasion, OMR = optimal margin region, RNA-seq = RNA sequencing, SDCH = Shangdong Cancer Hospital, TCGA = The Cancer Genome Atlas.

Between January 2023 and December 2024, 117 additional patients with HR-sHCC at Harbin Medical University Cancer Hospital were assessed for RNA sequencing. After excluding nine patients for poor imaging quality, seven for tumor diameter < 2 cm, 20 for incomplete clinical or pathologic information, 11 for a history of malignancy, and seven for gross vessel invasion at MRI, 63 patients remained, 30 of whom were included in the immunohistochemistry cohort. An additional 368 patients from TCGA were included in the TCGA cohort (Fig 1).

### Determining the Optimal Margin Region

**Figure 2:**
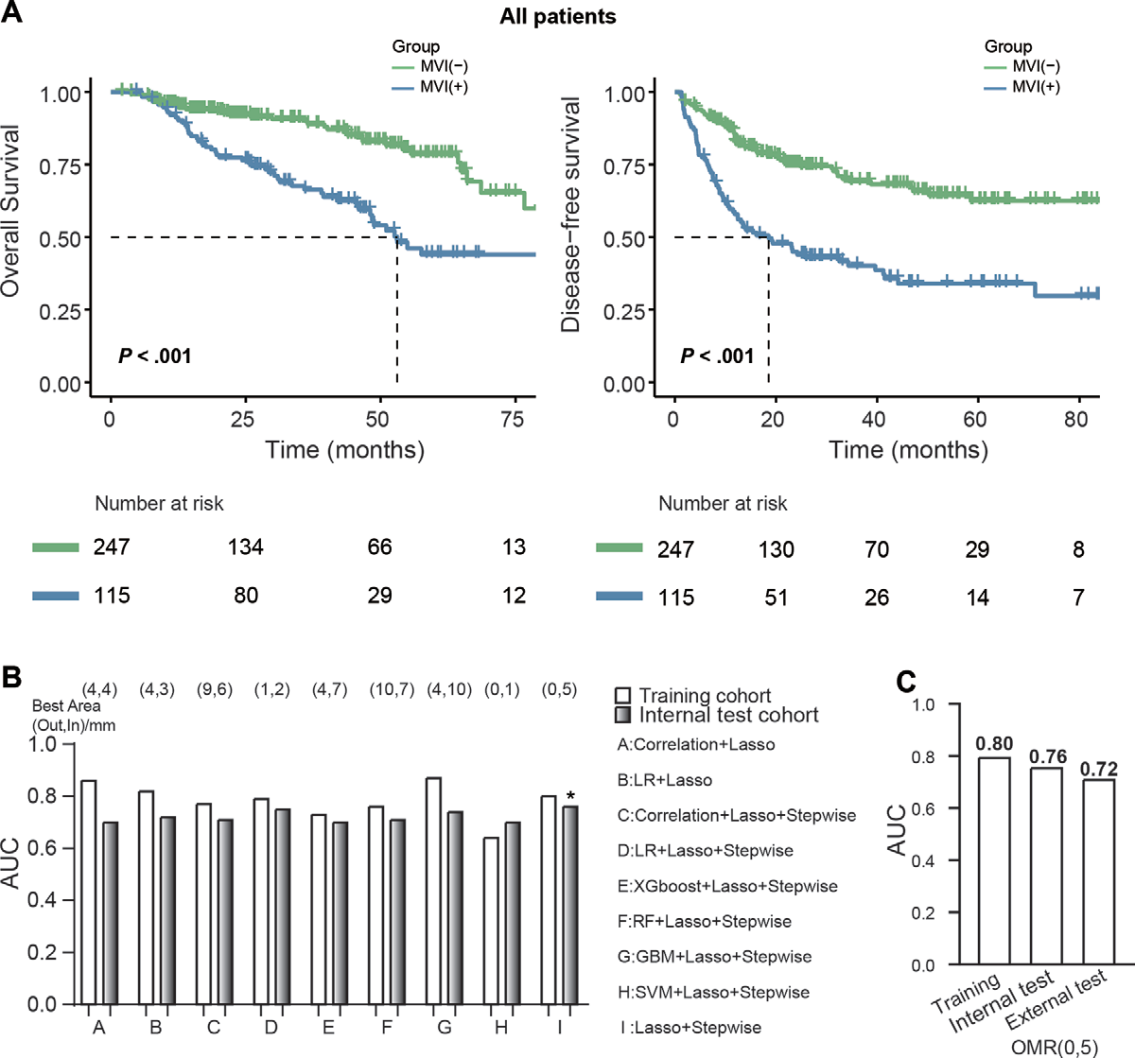
Selection of the optimal method combination and optimal margin region (OMR) and the performance of each model. **(A)** Survival curves of overall survival and disease-free survival for all patients whose tumors were positive or negative for microvascular invasion (MVI). **(B)** Among combinations of nine statistical and machine learning methods, the OMRs and area under the receiver operating characteristic curve (AUC) values in the training and internal test cohorts. **(C)** AUC values of OMRs in the training, internal test, and external test cohorts. GBM = gradient boosting machine, Lasso = least absolute shrinkage and selection operator, LR = logistic regression, RF = random forest, SVM = support vector machine, XGboost = extreme gradient boosting.

We confirmed the validity of MVI as a predictive clinical end point. For overall survival, median overall survival was 53.1 months (IQR, 48.1 months to not reached) for MVI positive versus not reached (IQR, 76.6 months to not reached) for MVI negative (log-rank *P* < .001). For disease-free survival, median disease-free survival was 18.5 months (IQR, 11.8–39.6 months) for MVI positive versus not reached (IQR, not reached to not reached) for MVI negative (log-rank *P* < .001) (Fig 2A). In the training cohort, we selected features from 121 VOIs by using nine different methods, resulting in 9 × 121 models. We generated AUC values for the training and internal test cohorts, and we determined the optimal method combination and optimal margin region on the basis of the highest AUC value in the internal test cohort. According to the maximum AUC value in the internal test cohort, we determined that the optimal method combination was LASSO + stepwise. We identified the corresponding optimal margin region as the outer 0–mm, inner 5–mm region after performing 10-fold cross-validation in the training cohort, which achieved AUC values of 0.80 (95% CI: 0.74, 0.86), 0.76 (95% CI: 0.66, 0.85), and 0.72 (95% CI: 0.58, 0.86) in the training, internal test, and external test cohorts, respectively (Fig 2B, 2C). After applying false discovery rate correction, the AUCs in the training, internal test, and external test cohorts were 0.76 (95% CI: 0.70, 0.83), 0.74 (95% CI: 0.64, 0.84), and 0.67 (95% CI: 0.53, 0.81), respectively. For comparison, the clinically established predictors α-fetoprotein, tumor size, and histologic grade achieved AUCs of 0.58 (95% CI: 0.51, 0.64), 0.63 (95% CI: 0.55, 0.70), and 0.60 (95% CI: 0.53, 0.67), respectively, in the training cohort (Table S3). Additionally, in the 121 internal training cohort models generated by the LASSO + stepwise method, six of the nine outer 0–mm regions were among the top eight regions with the highest AUC values, indicating a high likelihood of large AUC values for the outer 0–mm regions, whereas we did not observe this pattern in the outer 1–10–mm regions (Fig S2). Detailed performance metrics are provided in Table S4.

### Spatial Heterogeneity of HR-sHCC

To further explore the biologic basis of the selected optimal margin region and its relevance to tumor aggressiveness, we performed transcriptome sequencing analysis on tissue samples from 16 patients with HR-sHCC. We collected tissue samples from three distinct regions: the tumor core, the 5-mm tumor intratumoral margin, and the 5-mm tumor peritumoral margin. Although the increases in tumor intratumoral margin did not reach statistical significance (all *P* > .05), a consistent upward trend was observed across all indicators, which became more pronounced in the tumor peritumoral margin.

**Figure 3:**
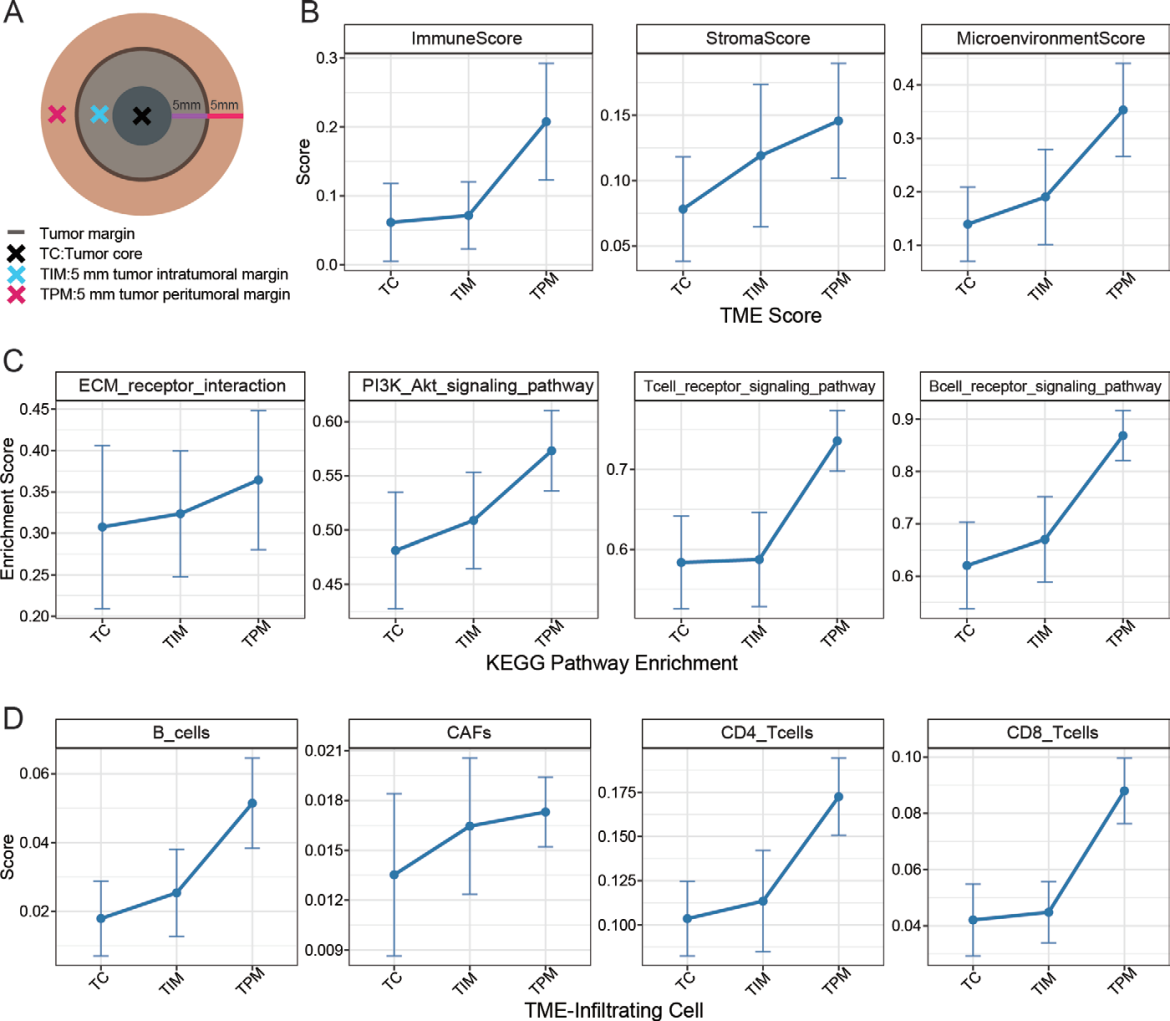
Spatial heterogeneity in high-risk solitary hepatocellular carcinoma reveals enhanced biologic activity at tumor margins. RNA sequencing was performed on three spatial regions: the tumor core (TC), the 5-mm tumor intratumoral margin (TIM), and the 5-mm tumor peritumoral margin (TPM). **(A)** Schematic diagram illustrates tissue sampling locations. **(B)** Tumor microenvironment (TME) scores across the three spatial regions. **(C)** Kyoto Encyclopedia of Genes and Genomes (KEGG) pathway enrichment analysis highlights differential pathway activity. **(D)** Analysis of TME-infiltrating immune cell populations in each region. CAF = cancer-associated fibroblast, ECM = extracellular matrix, PI3K = phosphatidylinositol 3-kinase.

For tumor microenvironment–related scores from xCell, tumor peritumoral margin exhibited higher ImmuneScore (0.06 ± 0.10 vs 0.21 ± 0.15, *P* = .003), StromaScore (0.08 ± 0.07 vs 0.15 ± 0.08, *P* = .014), and MicroenvironmentScore (0.14 ± 0.12 vs 0.35 ± 0.15, *P* < .001) compared with tumor core. Compared with tumor core, tumor peritumoral margin showed higher enrichment of the phosphatidylinositol 3-kinase (PI3K)–Akt signaling pathway (0.48 ± 0.09 vs 0.57 ± 0.06, *P* = .003), T-cell receptor signaling pathway (0.58 ± 0.10 vs 0.74 ± 0.07, *P* < .001), and B-cell receptor signaling pathway (0.62 ± 0.14 vs 0.87 ± 0.08, *P* < .001). Similarly, EPIC-based cell quantification revealed higher proportions of CD4^+^ T cells (0.10 ± 0.04 vs 0.17 ± 0.04, *P* < .01), CD8^+^ T cells (0.04 ± 0.02 vs 0.09 ± 0.02, *P* < .001), and B cells (0.02 ± 0.02 vs 0.05 ± 0.02, *P *< .001) in tumor peritumoral margin compared with tumor core (Fig 3).

### Tumor Margin Outer Region at MRI

According to the spatial heterogeneity results of HR-sHCC, regions inside and outside the tumor margin may be more suitable for predicting MVI; however, in our study, we defined the optimal margin region as the outer 0–mm, inner 5–mm region. Because of the limitations of MRI, this region often includes nontarget tissues (such as gallbladder, air, and vessels) in most patients, which compromises the stability of model feature learning (Fig S3). Under the 10-mm outward expansion, 427 of 436 patients (97.9%) included nontarget tissues in the expanded region. Further details are provided in Table 2.

**Table 2:** Summary of Nonhepatic Tissue Involvement in Expanded VOIs at Different Margins

Expansion (mm)	Liver Edge	Gallbladder	Portal Vein or Vessels	No. of New Patients Added
1	280	1	12	…
2	337	2	15	61
3	360	4	18	28
4	371	5	20	14
5	379	5	24	12
6	388	5	25	10
7	390	5	25	2
8	395	5	25	5
9	396	5	26	2
10	396	5	26	0

Note.—Values represent the numbers of patients in whom the expanded VOI at each specified margin (in millimeters) involved the corresponding nonhepatic tissue. *New patients added* indicates additional cases included compared with the previous expansion level. VOI = volume of interest.

### Radiomics Features from the Optimal Margin Region

The intra- and interreader consistency for VOI delineation was evaluated using the intraclass correlation coefficient. The seven selected radiomics features were logarithm_firstorder_Minimum, logarithm_glcm_Imc1, original_shape_Elongation, wavelet_LHL_glcm_MCC, wavelet_HLH_ngtdm_Busyness, log_sigma_3_0_mm_3D_glcm_ClusterShade, and wavelet_HHH_glcm_JointEntropy. Among them, original_shape_Elongation is related to tumor morphology, while the other features reflect signal characteristics at the tumor margin. The intraclass correlation coefficients of the seven features are detailed in Table S5. All seven features demonstrated intraclass correlation coefficient > 0.75, indicating their robustness for further analysis. We calculated SHapley Additive exPlanation values to determine the predictive role of each feature in the optimal radiomics model (Fig S4).

### Patient Stratification and Effectiveness

**Figure 4:**
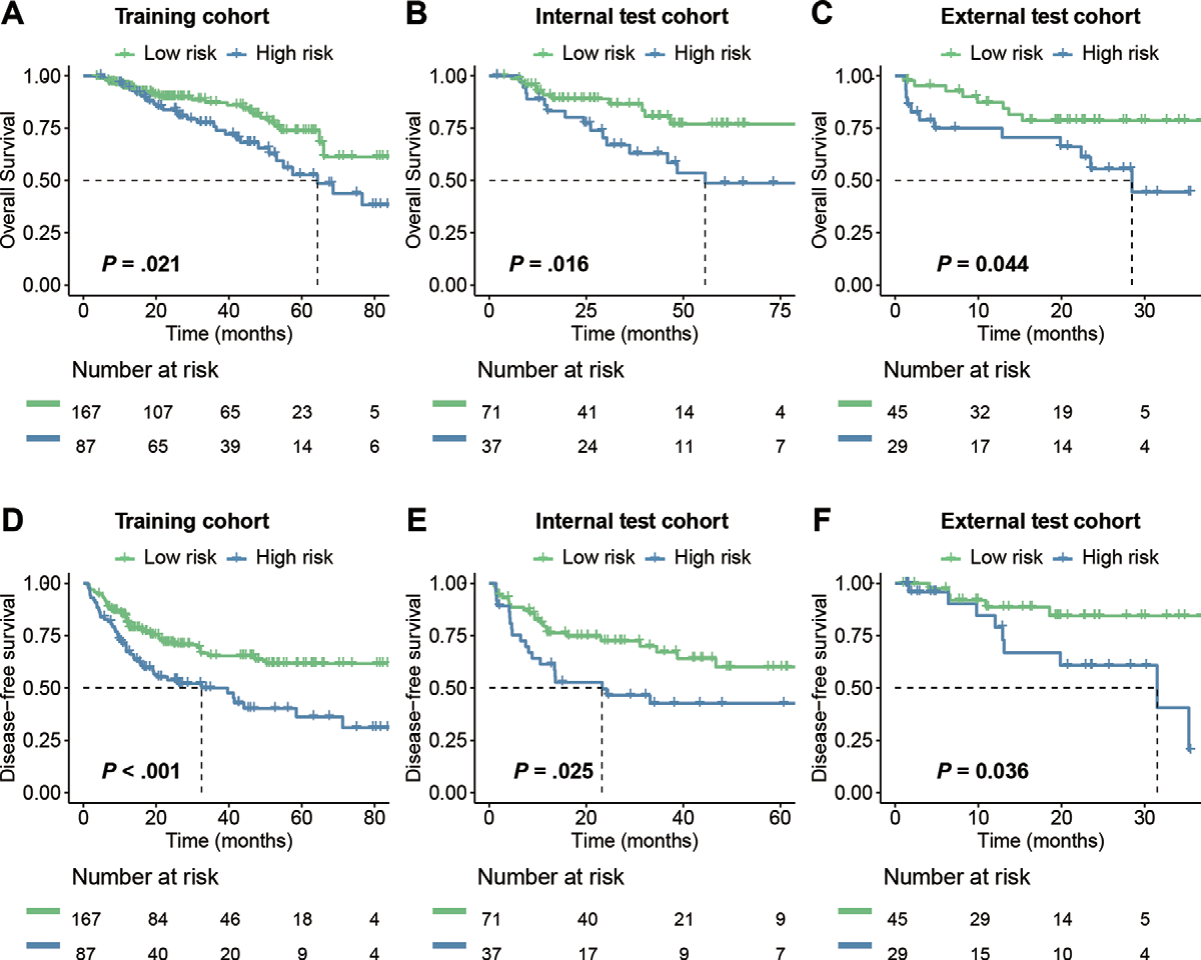
Survival curves for overall survival and disease-free survival for patient stratification with the model constructed according to the optimal method combination based on the optimal margin region in the **(A, D)** training group, **(B, E)** internal test group, and **(C, F)** external test group.

We combined the radiomics features from the optimal margin region into a radiomics score, and we stratified patients into optimal margin region high-risk and low-risk groups on the basis of the maximum Youden index. The performance of alternative thresholds is detailed in Table S6. We observed significant separation between the curves of the two patient groups in terms of overall survival and disease-free survival across the training, internal test, and external test cohorts (Fig 4, all* P* < .05). To further validate the rationale behind the radiomics-based stratification, we compared the radiologic features of the tumor margin between optimal margin region high-risk (*n* = 238) and low-risk patients (*n* = 124) from the Harbin Medical University Cancer Hospital center. The results, detailed in Table 3, revealed significant intergroup differences in peritumoral low signal and tumor morphology (*P* < .05). Specifically, the percentage of patients with peritumoral low signal was higher in the optimal margin region high-risk group (45 of 124, 36%) versus the optimal margin region low-risk group (50 of 238, 21%), while a higher percentage of patients in the optimal margin region low-risk group exhibited more regular tumor morphology (136 of 238, 57%) versus the optimal margin region high-risk group (50 of 124, 40%). The specific definitions of each radiologic feature are detailed in Table S7.

**Table 3:** Relationship between OMR Radiologic Features and OMR Groups

Characteristic	All Patients (*n* = 362)	OMR Low-Risk (*n* = 238)	OMR High-Risk (*n* = 124)	*P* Value
Peripheral washout				>.999
No	42 (11.6)	28 (11.8)	14 (11.3)	
Yes	320 (88.4)	210 (88.2)	110 (88.7)	
Capsule integrity				.192
No	154 (42.7)	93 (39.2)	61 (49.2)	
Yes	124 (34.3)	86 (36.3)	38 (30.6)	
No capsule	83 (23.0)	58 (24.5)	25 (20.2)	
Halo-like enhancement				.895
No	315 (87.0)	208 (87.4)	107 (86.3)	
Yes	47 (13.0)	30 (12.6)	17 (13.7)	
High Gd-EOB-DTPA uptake				.305
No	273 (75.4)	175 (73.5)	98 (79.0)	
Yes	89 (24.6)	63 (26.5)	26 (21.0)	
Peritumoral low signal				.003
No	267 (73.8)	188 (79.0)	79 (63.7)	
Yes	95 (26.2)	50 (21.0)	45 (36.3)	
Tumor morphology				.002
N	186 (51.5)	136 (57.4)	50 (40.3)	
CM	112 (31.0)	59 (24.9)	53 (42.7)	
NEG	63 (17.5)	42 (17.7)	21 (16.9)	

Note.—All data are presented as numbers, with percentages in parentheses. *P* values were calculated using the χ^2^ test or Fisher exact test, as appropriate, to compare categorical variables between OMR low-risk and high-risk groups. We considered a *P* value of less than .05 statistically significant. CM = nodular with extranodular growth, Gd-EOB-DTPA = gadolinium ethoxybenzyl-diethylenetriaminepentaacetic acid, N = nodular, NEG = confluent multinodular, OMR = optimal margin region.

### Biologic Functions Associated with the Optimal Margin Region Model

**Figure 5:**
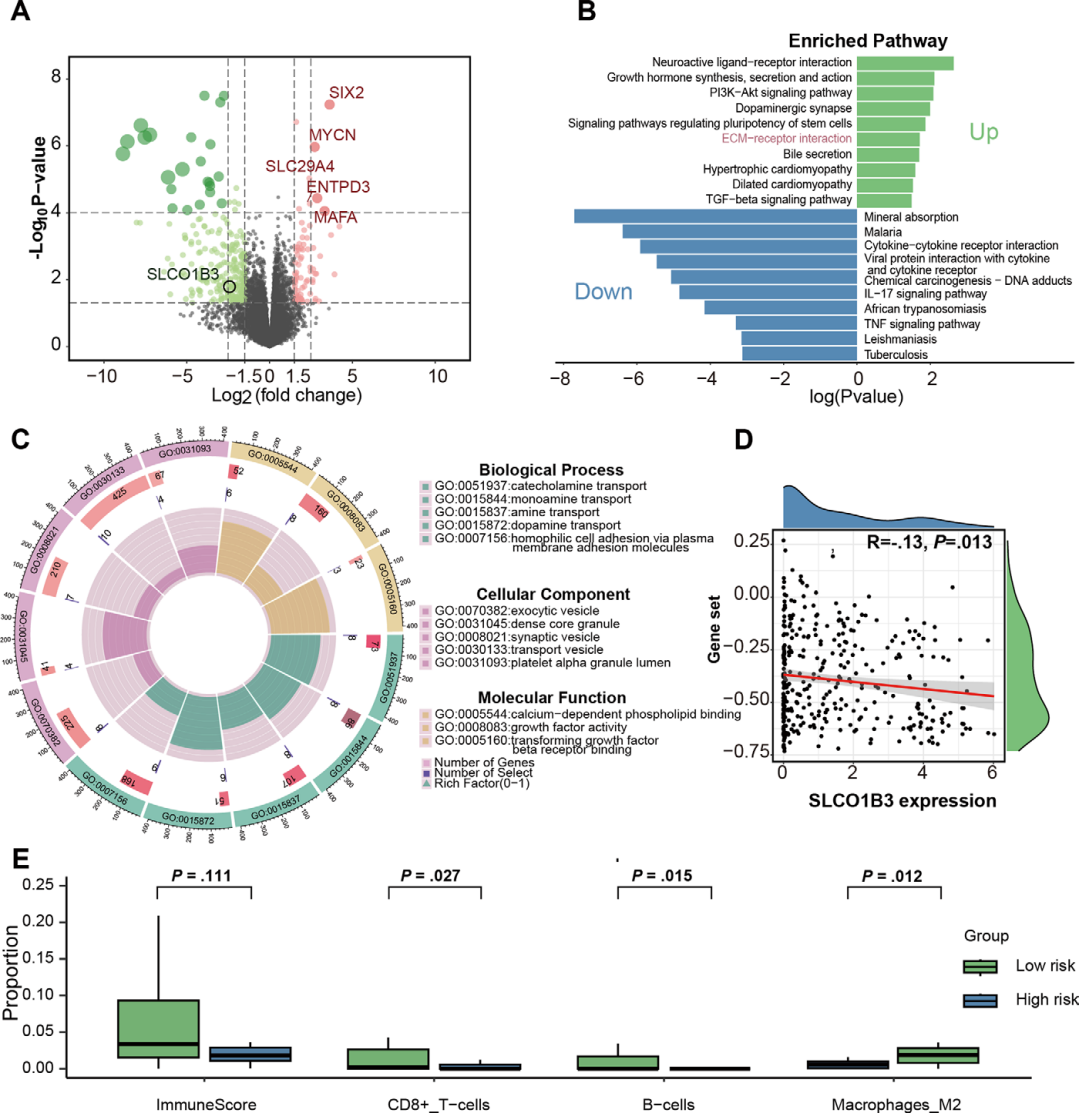
Patients were grouped according to the optimal margin region model based on RNA sequencing data. **(A)** Volcano plot shows differentially expressed genes between two patient groups, annotated with expression of *SLCO1B3* and the top five upregulated genes. **(B)** Kyoto Encyclopedia of Genes and Genomes pathway analysis of differentially expressed genes. **(C)** Gene Ontology (GO) analysis of differentially expressed genes. **(D)** Correlation analysis of the expression of the top five upregulated genes identified in The Cancer Genome Atlas dataset with *SLCO1B3* expression. **(E)** Immune infiltration box plots. ECM = extracellular matrix, PI3K = phosphatidylinositol 3-kinase, TGF = transforming growth factor, TNF = tumor necrosis factor.

We performed RNA sequencing of tissues from 63 patients (RNA-seq cohort), with patients grouped according to the model, resulting in 16 and 47 patients stratified into the optimal margin region high-risk and low-risk groups, respectively. We identified 515 differentially expressed genes, including 139 upregulated genes (Fig 5A). KEGG analysis of differentially expressed genes revealed extracellular matrix (ECM) receptor (*P* = .020), transforming growth factor (TGF)–β signaling (*P* = .032), and other pathways were enriched (Fig 5B). Gene Ontology analysis of the upregulated genes showed enrichment in homophilic cell adhesion via plasma membrane adhesion molecules (*P* < .001) and TGF-β receptor binding (*P* < .001), correlating with the enriched functions (Fig 5C). Compared with the optimal margin region high-risk group, ImmuneScore indicated that more immune cells were enriched in the low-risk group, with significant differences observed for CD8^+^ T cells (*P* = .027) and B cells (*P* = .015). However, M2 macrophages (*P* = .012) were more abundant in the high-risk group (Fig 5E). Our exploration of sensitivity to 237 commonly used anticancer drugs revealed significant differences in sensitivity to three drugs between the optimal margin region high-risk and low-risk groups (*P* < .05). Notably, the high-risk group exhibited higher sensitivity to MBS754807 and linsitinib, while the low-risk group showed higher sensitivity to tanespimycin (17-N-allylamino-17-demethoxygeldanamycin, or 17-AAG) (Fig S5).

### OATP1B3 Expression Association with Optimal Margin Region

**Figure 6:**
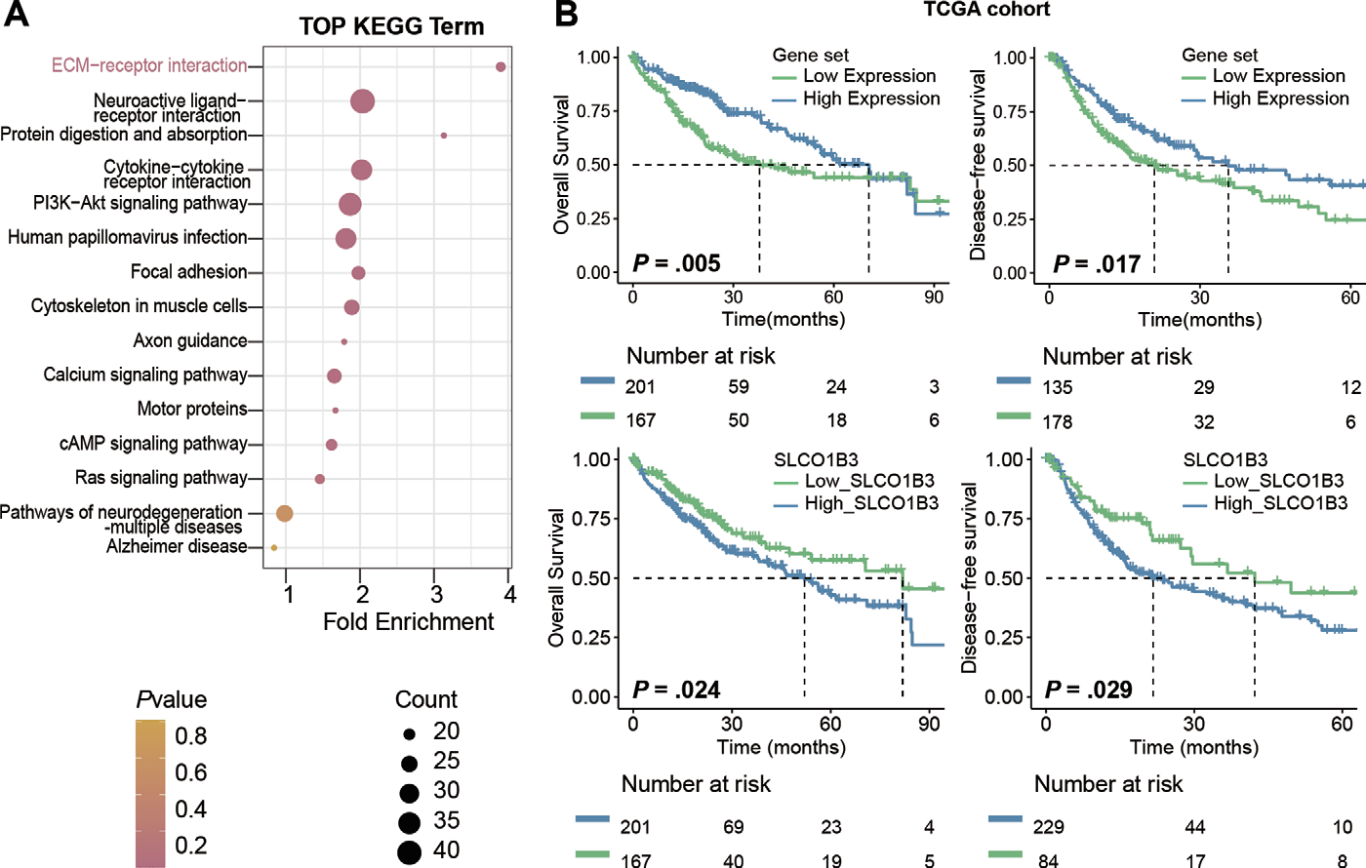
**(A)** Kyoto Encyclopedia of Genes and Genomes (KEGG) pathway analysis of patients in The Cancer Genome Atlas (TCGA) dataset stratified according to high and low expression levels of *SLCO1B3*. **(B)** Kaplan–Meier survival curves for overall survival and disease-free survival in the TCGA cohort, stratified by expression levels of *SLCO1B3* and a five-gene set (*SIX2, MYCN, SLC29A4, ENTPD3*, and *MAFA*). cAMP = cyclic adenosine monophosphate, ECM = extracellular matrix, PI3K = phosphatidylinositol 3-kinase.

OATP1B3 is the target protein for liver-specific contrast agents, making it important for study. The gene encoding OATP1B3 is *SLCO1B3*. Differential analysis of our sequencing data indicated that *SLCO1B3* expression was generally downregulated, whereas* SIX2, MYCN, SLC29A4, ENTPD3*, and* MAFA *(the five most heavily weighted upregulated genes) were upregulated in our optimal margin region high-risk patient group (Fig 5A). To further validate the relationship between *SLCO1B3* expression and patient risk stratification, we selected the five most heavily weighted upregulated genes as a gene set, and we used this gene set to compare its expression with RNA data from the TCGA cohort. We found that *SLCO1B3* expression was also negatively correlated with expression of this gene set in the TCGA cohort, with *P* < .05 (Fig 5D). We further explored OATP1B3 expression by grouping the TCGA cohort on the basis of *SLCO1B3* mean expression levels and using their fragments per kilobase of transcript per million mapped reads (or, FPKM) data, and then we performed differential analysis and KEGG clustering, which also revealed enrichment in ECM-receptor interaction pathways (Fig 6A). To assess the prognostic prediction capability of gene set and *SLCO1B3* expression, we performed log-rank tests on the overall survival and disease-free survival of patients in the TCGA cohort. For overall survival, median overall survival was 37.8 months (IQR, 22.9 months to not reached) for the high gene set expression group versus 70.5 months (IQR, 54.1 months to not reached) for the low gene set expression group (log-rank *P* = .005). For disease-free survival, median disease-free survival was 20.9 months (IQR, 15.0–40.4 months) for the high gene set expression group versus 35.6 months (IQR, 24.8 months to not reached) for the low gene set expression group (log-rank *P* = .017). (Fig 6B).

### Pseudocolor, Immunohistochemistry, and Tissue Staining

**Figure 7:**
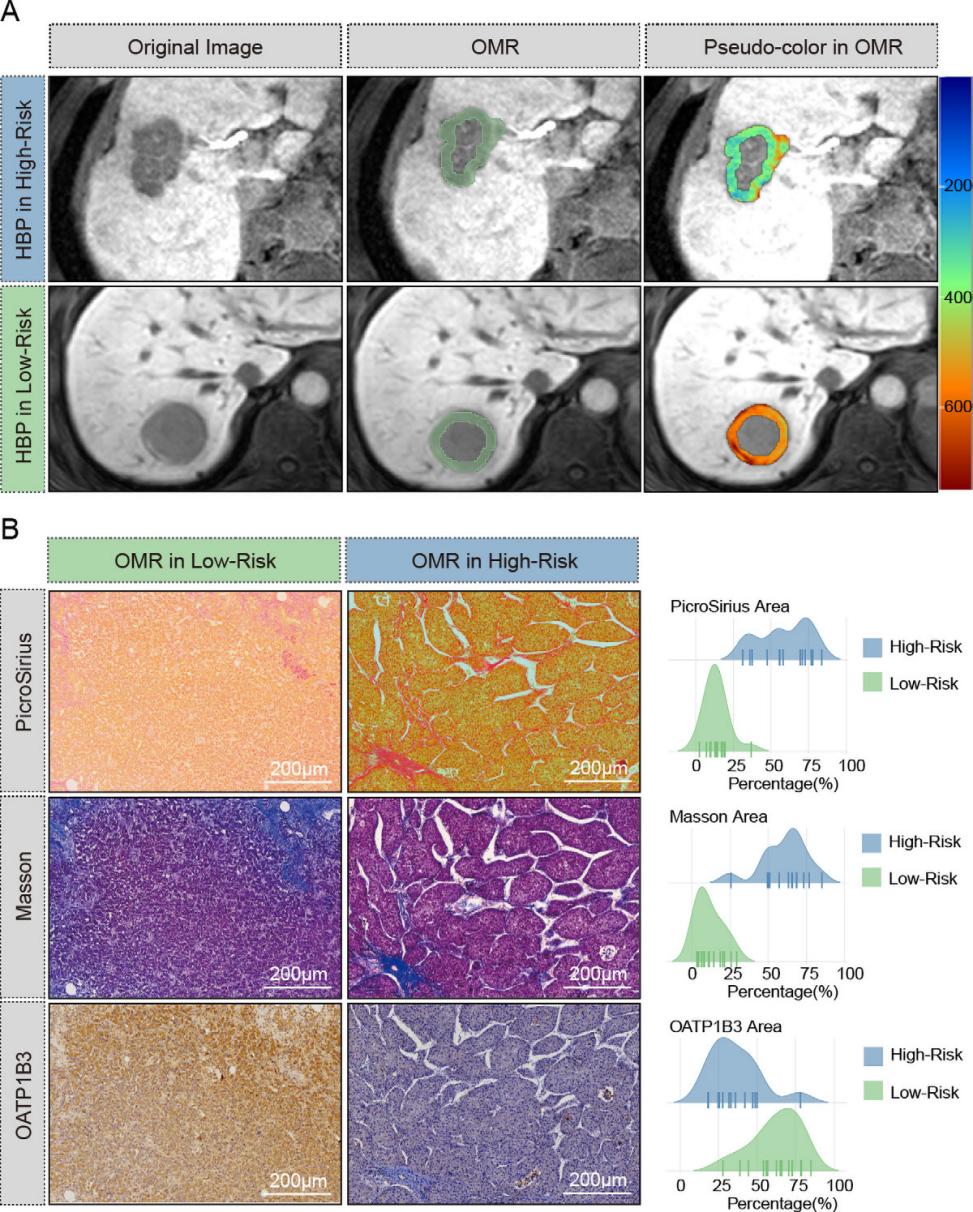
**(A)** Original images, optimal margin region (OMR), and pseudocolor images of the OMR in hepatobiliary phase (HBP) MRI for OMR high-risk and low-risk patient groups stratified according to the OMR model. The high-risk patient was male, 70 years old, microvascular invasion (MVI) positive, with recurrence detected 42 days after surgery and death 156 days after surgery. The low-risk patient was male, 66 years old, MVI negative, with recurrence and death within 5 years after surgery. **(B)** Microscopic views of PicroSirius red, Masson trichrome, and organic anion transporting polypeptide receptor 1B3 (OATP1B3) immunohistochemistry staining in high- and low-risk OMR groups, and mountain plots showing the percentage of stained area for each stain across 30 patients. All histochemical images were acquired at ×4 magnification.

Our results indicated that both the optimal margin region high-risk group in the RNA-seq cohort and the *SLCO1B3* low-expression group in the TCGA cohort enriched the ECM-receptor interaction pathway. To validate the reliability of the model in predicting ECM, we further stratified 30 patients (immunohistochemistry cohort) with HR-sHCC (14 optimal margin region high risk) from our institution using this model. Representative imaging from two patients is displayed, with pseudocolor visualization in the hepatobiliary phase. The low-risk patient exhibited more intense coloration, potentially indicating greater contrast agent uptake in the optimal margin region and higher OATP1B3 expression (Fig 7A).

Immunohistochemistry and quantitative analysis revealed more collagen fiber infiltration and lower OATP1B3 expression in the optimal margin region high-risk group tumors, whereas the margins of the optimal margin region low-risk group tumors had less collagen fiber and higher OATP1B3 expression than those of the optimal margin region high-risk group (Fig 7B).

## Discussion

MVI is a critical risk factor for postoperative tumor recurrence in patients with HCC, with the tumor margin identified as the primary site of invasion closely being linked to MVI development (28,29). In our study, we first identified the optimal margin region and developed a stable radiomics model for predicting MVI in HR-sHCC. The model showed robust performance (AUC: 0.80, 0.76, and 0.72 for training, internal test, and external test cohorts, respectively). Optimal margin region high-risk patients showed upregulation of a five-gene set (*SIX2*,* MYCN*,* SLC29A4*,* ENTPD3*, and* MAFA*), associated with shorter overall survival (37.8 vs 70.5 months, *P* = .005) and disease-free survival (20.9 vs 35.6 months, *P* = .017), whereas high *SLCO1B3* expression was linked to better outcomes (overall survival: 81.9 vs 52.0 months, *P* = .024; disease-free survival: 42.3 vs 21.6 months, *P* = .029). Immunohistochemistry findings confirmed that high-risk tumors had lower OATP1B3 expression and higher ECM deposition. These results indicate that the optimal margin region radiomics model robustly predicts survival differences and reflects underlying biologic heterogeneity. Clinically, delineating the optimal margin region provides a precise region of interest for evaluating the invasive potential at the tumor-liver interface. This noninvasive model can further assist in preoperative identification of patients at high risk of MVI, enabling more precise surgical and therapeutic planning. For low-risk patients, it may help avoid overly aggressive treatment, thereby preserving liver function and improving postoperative quality of life.

Although numerous studies have attempted to predict MVI in HCC preoperatively using multiphase CT (30,31), multiparametric MRI (32–34), or even cross-modal strategies that integrate clinical and imaging data (33–35), most have not systematically evaluated the potential value and underlying mechanisms of the tumor margin region. Wang et al (11) defined peritumoral regions by expanding outward from the tumor boundary by 5, 10, and 15 mm, yet such settings lack theoretical support. Through our study, we innovatively propose an optimal margin region selection strategy based on EOB MRI, systematically quantifying radiomics features within a 0- to 10-mm range inside and outside the tumor boundary. Ultimately, the outer 0–mm, inner 5–mm region was identified as the optimal margin region for MVI prediction. Compared with previously published models that primarily rely on intratumoral features or manually defined peritumoral margins—with reported AUCs typically ranging from 0.74 to 0.80—our optimal margin region–based radiomics model achieved relatively good performance, with AUCs of 0.76 and 0.72 in the internal test and external test cohorts, respectively (36,37). Benefiting from the biologic properties of EOB, the optimal margin region–based radiomics model offers greater biologic interpretability compared with conventional models. Moreover, when benchmarked against conventional clinical indicators such as α-fetoprotein (AUC = 0.58), tumor size (AUC = 0.63), and Edmondson grade (AUC = 0.60), the optimal margin region model showed higher discriminative ability.

For patients with HR-sHCC, simply using a fixed-width peritumoral region for feature extraction may overlook interindividual differences in surrounding anatomic structures, thereby limiting model robustness and interpretability. Our study also empirically deepens and addresses Liu et al’s (29) previous hypothesis that vessels and bile ducts around the tumor may increase regional heterogeneity and affect model performance. The proposed region selection strategy thus offers greater operability and provides new insights and methodologic avenues for MVI prediction research.

Among the radiomics features selected by the optimal margin region model, one feature was related to tumor morphology, while the other six were texture-related features that captured spatial variations in signal intensity and heterogeneity at the margin region. This finding aligns with the observed differences in radiologic features of tumor margins between the two groups, specifically in peritumoral low signal and tumor morphology. Previous research from our team has shown that more irregular and nonspherical tumors correlate with poorer prognoses (14,38), echoing our current findings. This study further revealed that the heterogeneity of tumor morphology and texture features within the optimal margin region represents another important factor contributing to the aggressiveness of HCC, visualizing its underlying histopathologic complexity (39). Such heterogeneity has been shown to be associated with unfavorable clinical outcomes (40,41). All these results highlight that the margin radiologic features were important indicators of MVI. Hepatobiliary phase images from EOB MRI improve tumor boundary visualization, likely influencing signal through the primary binding protein of EOB, OATP1B3 (42). The margin of HCC is known to contain a transition zone of normal hepatocytes and cancer-associated cells. The high-risk optimal margin region group exhibited low expression of *SLCO1B3* in our RNA sequencing analysis, suggesting less retention of normal hepatocytes, which was correlated with a worse prognosis and was consistent with the immunohistochemistry OATP1B3 expression results in tumor tissues. Taken together, the integration of morphologic and texture-based radiomic features with molecular evidence (eg, OATP1B3 expression) strengthens the biologic interpretability of the optimal margin region model and indicates that the imaging-derived heterogeneity indeed mirrors the underlying tumor biology at the invasive front. These findings reinforce the biologic relevance of our radiomics features.

According to the differential analysis results, *SIX2, MYCN, SLC29A4, ENTPD3*, and *MAFA* were the five most highly expressed genes in our high-risk group (43–47). Findings from previous studies suggested that these genes may be associated with tumor progression or tumor microenvironment regulation. With this gene set, we performed stratification analysis of patients in the TCGA cohort for outcomes and recurrence, and high expression of this gene set was associated with poorer patient outcomes. Enrichment analysis further indicated that the optimal margin region high-risk group showed elevated expression of proteins involved in signaling pathways such as TGF-β, ECM, and PI3K-Akt. The coexpression of these molecules and pathways suggests their potential synergistic role in the progression and invasion of HR-sHCC, consistent with previous research findings (48,49). A close relationship exists between MVI and the tumor microenvironment in the tumor margin region (50,51). Our optimal margin region model stratification results showed significant expression of ECM-related proteins in the optimal margin region of high-risk patients and in patients with low OATP1B3 expression. Further validation through immunohistochemistry and tissue staining confirmed the ECM protein expression levels in this region. Previous studies have shown that M2 macrophages promote ECM remodeling through TGF-β secretion, forming an immune barrier that prevents immune cell infiltration into the tumor (52). This process increases tumor invasiveness and contributes to poorer disease prognosis. In our study, we observed that high-risk patients exhibited greater ECM, TGF-β, and M2 macrophage presence within the optimal margin region, while immune cell infiltration was notably lower than that in low-risk patients. This finding provides new insights into the role of ECM-related immune barriers in MVI development, suggesting that they may create a more favorable microenvironment for tumor progression.

Our study had several limitations. First, its retrospective design may have introduced selection bias and limited generalizability. Second, the optimal margin region was identified in a data-driven manner, and although false discovery rate correction was applied to the final optimal margin region model, the initial evaluation of 121 candidate regions lacked multiple-testing correction, potentially increasing the risk of overfitting. Third, the relatively small external test cohort and intercenter variations in imaging protocols may have contributed to feature heterogeneity and affected model robustness. Fourth, the analysis focused solely on the hepatobiliary phase of EOB MRI without direct comparison to other MRI phases or CT, and deep learning approaches or established clinical predictors (eg, α-fetoprotein level, tumor size, histologic grade) were not incorporated. Finally, the biologic interpretation of radiomics features remains speculative and requires further experimental validation.

In conclusion, by applying multiple machine learning methods, we successfully identified the tumor margin region labeled as the outer 0 mm, inner 5 mm on hepatobiliary phase images as the optimal margin region, a critical area associated with the aggressiveness and prognosis of HR-sHCC. However, for HR-sHCC, applying a fixed and uniform margin region for radiomics feature extraction may not be an ideal or standardized approach. On the basis of this finding, we developed the first multicenter radiomics model capable of accurately predicting MVI in patients with HR-sHCC. Biologic interpretation of the model revealed that the expression of OATP1B3—a protein specific to EOB MRI—and components of the ECM within the optimal margin region serve as novel biomarkers for assessing HR-sHCC invasiveness and prognosis. Radiomics features extracted from the optimal margin region reflect the tumor microenvironment, with high-risk groups showing strong associations with M2 macrophage infiltration, activation of the TGF-β signaling pathway, and ECM-receptor interactions. This model enables effective stratification of patients with HR-sHCC and provides a valuable foundation for personalized therapeutic strategies.

## Supplemental Files

Tables S1-S7, Figures S1-S5

Conflicts of Interest
