# Supplementary material for: Gadoxetic Acid–enhanced MRI Radiomics Features of Tumor Margins for Predicting High-Risk Solitary Hepatocellular Carcinoma Aggressiveness and Prognosis
Source: Radiol Imaging Cancer. 2026 Jan 23;8(1):e250220. doi: 10.1148/rycan.250220 (PMC12862467; doi:10.1148/rycan.250220)
Supplement: Tables S1-S7, Figures S1-S5 [file rycan250220suppa1.pdf]

Supplemental materials undergo peer review but are not copyedited, and may include typographical errors.

©RSNA, 2026
10.1148/rycan.250220

Table S1: Hepatobiliary Phase (HBP) MRI Acquisition Parameters Across Study Centers.

**Study Center**	**HMUCH Center**	**HMUCH Center**	**HNCH Center**	**SDCH Center**
**MRI Vendor / Model**	Philips Ingenia 3.0 T	GE SIGNA Architect 3.0 T	Philips Ingenia CX 3.0 T	Siemens MAGNETOM Skyra 3.0 T
**TR (ms)**	3.6	4.7	3.7	3.97
**TE (ms)**	1.31	1.8	1.32	1.29
**FOV (mm²)**	400 × 333	400 × 340	400 × 384	380 × 320
**Matrix**	252 × 196	320 × 256	200 × 200	360 × 360
**Slice thickness (mm)**	4.0	2.5	4.0	6.0
**Slice gap (mm)**	−2*	0*	2.0	1.0
**Flip angle (°)**	15	20	9	9

*Note: Negative slice gap values indicate overlapping slices to ensure continuous 3D volume coverage, while 0 mm indicates contiguous slices without gaps.

Table S2: Statistical and Machine Learning Method: Definitions and Descriptions

Method	Definition
Correlation	A feature reduction technique where the correlation coefficient matrix of features in the training cohort is calculated. Features with absolute correlation coefficients greater than 0.8 are considered highly correlated and are removed to avoid multicollinearity and improve model performance.
LASSO (Least Absolute Shrinkage and Selection Operator)	A regularization method used for feature reduction in regression models. It involves penalizing the absolute size of regression coefficients, which can drive some coefficients to zero, effectively performing feature selection. The hyperparameter λ (lambda) controls the strength of the penalty. In this study, λ was chosen using the 1-standard error criterion based on five-fold cross-validation, which balances model complexity and performance by selecting a λ that is within one standard error of the minimum cross-validation error.
LR (Logistic regression)	A statistical model used for binary classification tasks that models the probability of a binary outcome based on one or more predictor variables. It uses the logistic function to

Supplemental materials undergo peer review but are not copyedited, and may include typographical errors.

	output a probability that is mapped to a binary outcome (0 or 1).
Stepwise	A method for selecting a subset of predictor variables for use in a regression model. It involves adding or removing predictors based on criteria such as the Akaike Information Criterion (AIC) or Bayesian Information Criterion (BIC) to identify the most suitable model. In this study, the AIC criterion was used to determine which predictors to include or exclude, aiming to find a model that best balances goodness-of-fit with model complexity.
XGBoost (Extreme Gradient Boosting)	A scalable and efficient implementation of gradient boosting that is widely used for supervised learning tasks. It builds an ensemble of weak learners (typically decision trees) and optimizes their performance by minimizing a specified loss function. In this study, we selected the top 30 most important features based on their weights for subsequent analysis.
RF (Random Forest)	An extension of random forests used for analyzing binary outcomes. It provides a non-parametric approach to classification and identifying important variables. In this study, we selected the top 30 most important features based on their weights for subsequent analysis.
GBM (Gradient Boosting Machine)	An ensemble technique that builds models sequentially, where each model attempts to correct the errors of the previous one. It combines multiple weak learners (usually decision trees) to produce a robust predictive model by optimizing a loss function. In this study, we selected the top 30 most important features based on their weights for subsequent analysis.
SVM (Support Vector Machine)	A supervised learning algorithm used for classification and regression tasks. It finds the hyperplane that best separates different classes in the feature space by maximizing the margin between them. It can handle both linear and non-linear relationships through the use of kernel functions. In this study, we selected the top 30 most important features based on their weights for subsequent analysis.

Table S3: AUC for AFP, tumor size, and histological grade in predicting MVI in the training, internal test, and external testing cohorts.

**Characteristic**	**Train cohort**	**Valid cohort**	**External cohort**
AFP	0.58	0.57	0.49
Size	0.63	0.64	0.65
Histological grade	0.60	0.61	0.55

Supplemental materials undergo peer review but are not copyedited, and may include typographical errors.

AUC, Area under the receiver operating characteristic curve; MVI, microvascular invasion.

Table S4: Summary of Radiomic Features and Model Performance Using LASSO and Stepwise Regression

Region (outer mar inner) (mm)	Training Cohort AUC	Internal Test AUC	Number of Features	Radiomic Features
0mar1	0.79	0.61	6	log_sigma_3_0_mm_3D_glszm_LargeAreaHighGrayLevelEmphasis<br>log_sigma_4_0_mm_3D_firstorder_Skewness<br>log_sigma_4_0_mm_3D_glszm_GrayLevelNonUniformity<br>square_gldm_LargeDependenceHighGrayLevelEmphasis<br>wavelet_HHL_firstorder_Kurtosis<br>wavelet_HHL_glcm_JointEnergy
0mar2	0.77	0.63	4	logarithm_firstorder_Minimum<br>logarithm_gldm_LargeDependenceEmphasis<br>wavelet_HLH_ngtdm_Busyness<br>wavelet_HLL_glcm_MCC
0mar3	0.76	0.64	5	logarithm_firstorder_Minimum<br>logarithm_glcm_Imc1<br>wavelet_HLH_ngtdm_Busyness<br>wavelet_HLL_firstorder_Skewness<br>wavelet_LHL_gldm_LargeDependenceLowGrayLevelEmphasis
0mar4	0.83	0.71	10	exponential_glszm_SmallAreaEmphasis<br>gradient_firstorder_Skewness<br>log_sigma_3_0_mm_3D_glcm_ClusterShade<br>log_sigma_4_0_mm_3D_gldm_LargeDependenceHighGrayLevelEmphasis<br>logarithm_firstorder_Minimum<br>original_shape_Elongation<br>wavelet_HHH_glcm_JointEntropy<br>wavelet_HLH_ngtdm_Busyness<br>wavelet_LHL_glcm_MCC

Supplemental materials undergo peer review but are not copyedited, and may include typographical errors.

				wavelet_LHL_gldm_LargeDependenceLowGrayLevelEmphasis
0mar5	0.80	0.76	7	log_sigma_3_0_mm_3D_glcm_ClusterShade<br>logarithm_firstorder_Minimum<br>logarithm_glcm_Imc1<br>original_shape_Elongation<br>wavelet_HHH_glcm_JointEntropy<br>wavelet_HLH_ngtdm_Busyness<br>wavelet_LHL_glcm_MCC
0mar6	0.81	0.74	9	log_sigma_3_0_mm_3D_glcm_ClusterShade<br>log_sigma_5_0_mm_3D_gldm_LargeDependenceHighGrayLevelEmphasis<br>logarithm_firstorder_Minimum<br>logarithm_glcm_Imc1<br>original_shape_Elongation<br>wavelet_HHH_glcm_JointEnergy<br>wavelet_HLH_ngtdm_Busyness<br>wavelet_HLL_firstorder_Skewness<br>wavelet_LHL_glcm_MCC
0mar7	0.77	0.69	6	log_sigma_5_0_mm_3D_gldm_LargeDependenceHighGrayLevelEmphasis<br>logarithm_firstorder_Minimum<br>original_shape_Elongation<br>wavelet_HHH_glcm_JointEntropy<br>wavelet_HLH_ngtdm_Busyness<br>wavelet_HLL_glcm_MCC
0mar8	0.79	0.67	7	log_sigma_5_0_mm_3D_gldm_LargeDependenceHighGrayLevelEmphasis<br>logarithm_firstorder_Minimum<br>original_shape_Elongation<br>wavelet_HHH_glcm_JointEntropy<br>wavelet_HLH_ngtdm_Busyness<br>wavelet_HLL_glcm_MCC<br>wavelet_LHL_gldm_LargeDependenceLowGrayLevelEmphasis
0mar9	0.77	0.70	6	log_sigma_5_0_mm_3D_gldm_LargeDependenceHighGrayLevelEmphasis

Supplemental materials undergo peer review but are not copyedited, and may include typographical errors.

				original_shape_Elongation<br>squareroot_firstorder_Minimum<br>wavelet_HHH_glcm_JointEntropy<br>wavelet_HLH_ngtdm_Busyness<br>wavelet_HLL_glcm_MCC
0mar10	0.77	0.70	6	log_sigma_5_0_mm_3D_gldm_LargeDependenceHighGrayLevelEmphasis<br>original_shape_Elongation<br>squareroot_firstorder_Minimum<br>wavelet_HHH_glcm_JointEntropy<br>wavelet_HLH_ngtdm_Busyness<br>wavelet_HLL_glcm_MCC
1mar0	0.79	0.60	6	lbp_3D_m2_firstorder_Kurtosis<br>log_sigma_5_0_mm_3D_firstorder_Kurtosis<br>square_gldm_LargeDependenceLowGrayLevelEmphasis<br>squareroot_gldm_LargeDependenceEmphasis<br>wavelet_HLH_ngtdm_Busyness<br>wavelet_HLL_glcm_MCC
1mar1	0.78	0.57	8	lbp_3D_m2_glszm_SmallAreaEmphasis<br>original_shape_Elongation<br>squareroot_gldm_DependenceVariance<br>wavelet_HHL_glcm_ClusterShade<br>wavelet_HLH_firstorder_Skewness<br>wavelet_HLL_glcm_MCC<br>wavelet_LHH_gldm_SmallDependenceEmphasis<br>wavelet_LHH_glszm_LargeAreaLowGrayLevelEmphasis
1mar2	0.78	0.62	5	logarithm_glcm_DifferenceVariance<br>logarithm_gldm_LargeDependenceEmphasis<br>original_shape_Elongation<br>wavelet_HLH_ngtdm_Busyness<br>wavelet_HLL_glcm_MCC

Supplemental materials undergo peer review but are not copyedited, and may include typographical errors.

1mar3	0.80	0.65	7	logarithm_glcm_DifferenceVariance<br>logarithm_gldm_LargeDependenceEmphasis<br>original_shape_Elongation<br>wavelet_HHH_glszm_SizeZoneNonUniformityNormalized<br>wavelet_HHL_ngtdm_Strength<br>wavelet_HLH_ngtdm_Busyness<br>wavelet_HLL_glcm_MCC
1mar4	0.77	0.63	5	logarithm_glcm_DifferenceVariance<br>original_shape_Elongation<br>squareroot_glcm_JointEnergy<br>wavelet_HLH_ngtdm_Busyness<br>wavelet_HLL_glcm_MCC
1mar5	0.78	0.63	6	lbp_2D_glszm_GrayLevelNonUniformityNormalized<br>logarithm_glcm_DifferenceVariance<br>original_shape_Elongation<br>squareroot_glcm_JointEnergy<br>wavelet_HLH_ngtdm_Busyness<br>wavelet_HLL_glcm_MCC
1mar6	0.78	0.62	6	gradient_ngtdm_Strength<br>logarithm_glcm_DifferenceVariance<br>logarithm_gldm_LargeDependenceEmphasis<br>original_shape_Elongation<br>wavelet_HLH_ngtdm_Busyness<br>wavelet_HLL_glcm_MCC
1mar7	0.79	0.65	7	gradient_ngtdm_Strength<br>log_sigma_3_0_mm_3D_firstorder_Kurtosis<br>logarithm_glcm_DifferenceVariance<br>original_shape_Elongation<br>squareroot_glcm_JointEnergy<br>wavelet_HLH_ngtdm_Busyness<br>wavelet_HLL_glcm_MCC
1mar8	0.78	0.64	6	gradient_ngtdm_Strength<br>logarithm_glcm_DifferenceVariance<br>original_shape_Elongation

Supplemental materials undergo peer review but are not copyedited, and may include typographical errors.

				squareroot_glcm_JointEnergy<br>wavelet_HLH_ngtdm_Busyness<br>wavelet_HLL_glcm_MCC
1mar9	0.72	0.61	5	logarithm_glcm_DifferenceVariance<br>logarithm_gldm_LargeDependenceEmphasis<br>original_shape_Elongation<br>wavelet_HHH_glcm_JointEnergy<br>wavelet_HLL_glcm_MCC
1mar10	0.78	0.63	6	lbp_2D_glszm_GrayLevelNonUniformityNormalized<br>logarithm_glcm_DifferenceVariance<br>original_shape_Elongation<br>squareroot_glcm_JointEnergy<br>wavelet_HLH_ngtdm_Busyness<br>wavelet_HLL_glcm_MCC
2mar0	0.71	0.57	3	lbp_2D_firstorder_10Percentile<br>log_sigma_5_0_mm_3D_firstorder_Kurtosis<br>square_gldm_LargeDependenceLowGrayLevelEmphasis
2mar1	0.85	0.65	11	gradient_firstorder_Skewness<br>lbp_3D_k_firstorder_Kurtosis<br>lbp_3D_m2_firstorder_Kurtosis<br>log_sigma_5_0_mm_3D_firstorder_Kurtosis<br>original_firstorder_10Percentile<br>original_shape_Elongation<br>squareroot_gldm_DependenceVariance<br>wavelet_HHH_glszm_ZoneEntropy<br>wavelet_HLH_ngtdm_Busyness<br>wavelet_HLL_glcm_MCC<br>wavelet_LHH_gldm_SmallDependenceEmphasis
2mar2	0.50	0.50	0	
2mar10	0.59	0.53	1	squareroot_glcm_MaximumProbability

Supplemental materials undergo peer review but are not copyedited, and may include typographical errors.

2mar3	0.85	0.66	11	lbp_3D_k_firstorder_Kurtosis<br>lbp_3D_m2_firstorder_Kurtosis<br>log_sigma_5_0_mm_3D_firstorder_Kurtosis<br>original_shape_Elongation<br>squareroot_gldm_LargeDependenceEmphasis<br>wavelet_HHH_glszm_LowGrayLevelZoneEmphasis<br>wavelet_HLH_glszm_SizeZoneNonUniformityNormalized<br>wavelet_HLH_ngtdm_Busyness<br>wavelet_LHH_gldm_SmallDependenceEmphasis<br>wavelet_LHL_glcm_MCC<br>wavelet_LLL_firstorder_10Percentile
2mar4	0.50	0.50	0	
2mar5	0.69	0.45	2	lbp_3D_m1_gldm_DependenceEntropy<br>squareroot_glcm_MaximumProbability
2mar6	0.69	0.46	2	lbp_3D_m1_gldm_DependenceEntropy<br>squareroot_glcm_MaximumProbability
2mar7	0.86	0.62	9	gradient_firstorder_Skewness<br>lbp_3D_k_firstorder_Kurtosis<br>lbp_3D_m1_gldm_DependenceEntropy<br>log_sigma_3_0_mm_3D_firstorder_Kurtosis<br>squareroot_glcm_JointEnergy<br>wavelet_HHH_glszm_LowGrayLevelZoneEmphasis<br>wavelet_HLH_gldm_LowGrayLevelEmphasis<br>wavelet_LHL_glcm_ClusterProminence<br>wavelet_LLL_firstorder_10Percentile
2mar8	0.85	0.62	9	gradient_firstorder_Skewness

Supplemental materials undergo peer review but are not copyedited, and may include typographical errors.

				lbp_3D_k_firstorder_Kurtosis<br>lbp_3D_m1_gldm_DependenceEntropy<br>log_sigma_3_0_mm_3D_firstorder_Kurtosis<br>original_firstorder_10Percentile<br>squareroot_glcm_JointEnergy<br>wavelet_HHH_glszm_LowGrayLevelZoneEmphasis<br>wavelet_HLH_gldm_LowGrayLevelEmphasis<br>wavelet_LHL_glcm_ClusterProminence
2mar9	0.66	0.53	1	lbp_3D_m1_gldm_DependenceEntropy
2mar10	0.59	0.53	1	squareroot_glcm_MaximumProbability
3mar0	0.79	0.60	6	lbp_3D_m1_gldm_DependenceEntropy<br>lbp_3D_m2_firstorder_Kurtosis<br>log_sigma_4_0_mm_3D_firstorder_Kurtosis<br>squareroot_glcm_MaximumProbability<br>wavelet_HLH_ngtdm_Busyness<br>wavelet_HLL_glcm_MCC
3mar1	0.86	0.63	11	gradient_firstorder_Skewness<br>lbp_3D_k_firstorder_Kurtosis<br>lbp_3D_m2_firstorder_Kurtosis<br>log_sigma_4_0_mm_3D_firstorder_Kurtosis<br>log_sigma_5_0_mm_3D_ngtdm_Contrast<br>logarithm_glcm_Idmn<br>original_shape_Elongation<br>squareroot_glcm_MaximumProbability<br>wavelet_HLH_firstorder_Skewness<br>wavelet_HLH_ngtdm_Contrast<br>wavelet_HLL_glcm_MCC

Supplemental materials undergo peer review but are not copyedited, and may include typographical errors.

3mar2	0.80	0.59	7	gradient_firstorder_Skewness<br>lbp_3D_m1_gldm_DependenceEntropy<br>lbp_3D_m2_firstorder_Kurtosis<br>log_sigma_5_0_mm_3D_firstorder_Kurtosis<br>squareroot_gldm_LargeDependenceEmphasis<br>wavelet_HLH_ngtdm_Busyness<br>wavelet_HLL_glcm_MCC
3mar3	0.71	0.54	2	lbp_3D_m1_gldm_DependenceEntropy<br>squareroot_glcm_MaximumProbability
3mar4	0.70	0.53	2	lbp_3D_m1_gldm_DependenceEntropy<br>squareroot_glcm_MaximumProbability
3mar5	0.70	0.48	2	lbp_3D_m1_gldm_DependenceEntropy<br>squareroot_glcm_MaximumProbability
3mar6	0.70	0.56	2	lbp_3D_m1_gldm_DependenceEntropy<br>square_ngtdm_Busyness
3mar7	0.70	0.56	2	lbp_3D_m1_gldm_DependenceEntropy<br>square_ngtdm_Busyness
3mar8	0.70	0.49	2	lbp_3D_m1_gldm_DependenceEntropy<br>squareroot_glcm_MaximumProbability
3mar9	0.69	0.56	2	lbp_3D_m1_gldm_DependenceEntropy<br>square_ngtdm_Busyness

Supplemental materials undergo peer review but are not copyedited, and may include typographical errors.

3mar10	0.69	0.55	2	lbp_3D_m1_gldm_DependenceEntropy<br>square_ngtdm_Busyness
4mar0	0.75	0.70	4	lbp_3D_k_firstorder_Kurtosis<br>lbp_3D_m2_glrlm_HighGrayLevelRunEmphasis<br>log_sigma_4_0_mm_3D_ngtdm_Contrast<br>square_gldm_LargeDependenceLowGrayLevelEmphasis
4mar1	0.87	0.68	11	gradient_glcm_MaximumProbability<br>lbp_3D_k_firstorder_Kurtosis<br>lbp_3D_m2_firstorder_Kurtosis<br>log_sigma_3_0_mm_3D_firstorder_Kurtosis<br>log_sigma_5_0_mm_3D_ngtdm_Contrast<br>original_shape_Elongation<br>square_ngtdm_Busyness<br>squareroot_firstorder_10Percentile<br>wavelet_HHH_glszm_ZoneEntropy<br>wavelet_HHL_glcm_MCC<br>wavelet_HLH_firstorder_Skewness
4mar2	0.86	0.68	12	gradient_firstorder_Skewness<br>lbp_3D_k_firstorder_Kurtosis<br>lbp_3D_m2_firstorder_Kurtosis<br>log_sigma_3_0_mm_3D_firstorder_Kurtosis<br>log_sigma_4_0_mm_3D_firstorder_Kurtosis<br>log_sigma_5_0_mm_3D_ngtdm_Contrast<br>logarithm_glcm_Idmn<br>squareroot_gldm_DependenceVariance<br>wavelet_HLH_firstorder_Skewness<br>wavelet_HLH_glszm_LargeAreaLowGrayLevelEmphasis<br>wavelet_HLL_firstorder_Mean<br>wavelet_LHH_firstorder_Median
4mar3	0.70	0.58	2	log_sigma_5_0_mm_3D_ngtdm_Cont

Supplemental materials undergo peer review but are not copyedited, and may include typographical errors.

				rast<br>squareroot_glcm_MaximumProbability
4mar4	0.69	0.53	2	squareroot_glcm_MaximumProbability<br>wavelet_LHH_firstorder_Median
4mar5	0.70	0.59	2	log_sigma_5_0_mm_3D_ngtdm_Contrast<br>squareroot_glcm_MaximumProbability
4mar6	0.73	0.59	3	lbp_3D_m1_gldm_DependenceEntropy<br>log_sigma_5_0_mm_3D_ngtdm_Contrast<br>squareroot_glcm_MaximumProbability
4mar7	0.73	0.59	3	lbp_3D_m1_gldm_DependenceEntropy<br>log_sigma_5_0_mm_3D_ngtdm_Contrast<br>squareroot_glcm_MaximumProbability
4mar8	0.72	0.62	2	lbp_3D_m1_gldm_DependenceEntropy<br>log_sigma_5_0_mm_3D_ngtdm_Contrast
4mar9	0.66	0.62	1	lbp_3D_k_firstorder_Kurtosis
4mar10	0.71	0.62	2	lbp_3D_m1_gldm_DependenceEntropy<br>log_sigma_5_0_mm_3D_ngtdm_Contrast
5mar0	0.76	0.68	5	lbp_3D_k_firstorder_Kurtosis<br>lbp_3D_m2_glszm_GrayLevelVariance<br>log_sigma_4_0_mm_3D_glcm_Idmn<br>square_gldm_LargeDependenceLowG

Supplemental materials undergo peer review but are not copyedited, and may include typographical errors.

				rayLevelEmphasis<br>wavelet_HLH_glszm_SmallAreaEmphasis
5mar1	0.84	0.61	9	lbp_3D_k_firstorder_Kurtosis<br>lbp_3D_m2_firstorder_Kurtosis<br>log_sigma_2_0_mm_3D_glcm_ClusterShade<br>log_sigma_4_0_mm_3D_glszm_ZoneEntropy<br>log_sigma_4_0_mm_3D_ngtdm_Contrast<br>logarithm_ngtdm_Contrast<br>squareroot_glszm_LargeAreaHighGrayLevelEmphasis<br>wavelet_HHL_glcm_Imc2<br>wavelet_HLH_firstorder_Skewness
5mar2	0.82	0.64	8	lbp_3D_k_firstorder_Kurtosis<br>lbp_3D_m2_firstorder_Kurtosis<br>log_sigma_5_0_mm_3D_ngtdm_Contrast<br>logarithm_firstorder_RobustMeanAbsoluteDeviation<br>original_shape_Elongation<br>squareroot_glszm_LargeAreaHighGrayLevelEmphasis<br>wavelet_HHL_glcm_Imc2<br>wavelet_HLH_firstorder_Skewness
5mar3	0.83	0.64	8	lbp_3D_k_firstorder_Kurtosis<br>lbp_3D_m2_firstorder_Kurtosis<br>log_sigma_5_0_mm_3D_ngtdm_Contrast<br>logarithm_firstorder_RobustMeanAbsoluteDeviation<br>original_shape_Elongation<br>squareroot_glszm_LargeAreaHighGrayLevelEmphasis<br>wavelet_HHL_glcm_Imc2<br>wavelet_HLH_glszm_SizeZoneNonUniformityNormalized
5mar4	0.83	0.67	9	lbp_3D_k_firstorder_Kurtosis

Supplemental materials undergo peer review but are not copyedited, and may include typographical errors.

				lbp_3D_m2_firstorder_Kurtosis<br>log_sigma_4_0_mm_3D_glszm_ZoneEntropy<br>log_sigma_4_0_mm_3D_ngtdm_Contrast<br>logarithm_glcm_Idmn<br>original_shape_Elongation<br>squareroot_glszm_LargeAreaHighGrayLevelEmphasis<br>wavelet_HHL_glcm_Imc2<br>wavelet_HLH_glszm_SizeZoneNonUniformityNormalized
5mar5	0.76	0.66	5	lbp_3D_k_firstorder_Kurtosis<br>lbp_3D_m2_glrlm_HighGrayLevelRunEmphasis<br>original_shape_Elongation<br>square_glrlm_LongRunLowGrayLevelEmphasis<br>wavelet_HLH_glszm_SmallAreaEmphasis
5mar6	0.75	0.61	4	lbp_3D_k_firstorder_Kurtosis<br>original_shape_Elongation<br>squareroot_glcm_MaximumProbability<br>wavelet_HLH_glszm_SizeZoneNonUniformityNormalized
5mar7	0.75	0.62	4	lbp_3D_k_firstorder_Kurtosis<br>original_shape_Elongation<br>squareroot_glcm_MaximumProbability<br>wavelet_HLH_glszm_SizeZoneNonUniformityNormalized
5mar8	0.74	0.61	4	lbp_3D_k_firstorder_Kurtosis<br>lbp_3D_m1_gldm_DependenceEntropy<br>original_shape_Elongation<br>wavelet_HLH_glszm_SmallAreaEmphasis
5mar9	0.72	0.48	4	lbp_3D_m1_gldm_DependenceEntrop

Supplemental materials undergo peer review but are not copyedited, and may include typographical errors.

				y<br>original_shape_Elongation<br>squareroot_glcm_MaximumProbability<br>wavelet_HLH_glszm_SizeZoneNonUniformityNormalized
5mar10	0.76	0.66	5	lbp_3D_k_firstorder_Kurtosis<br>lbp_3D_m2_glrlm_HighGrayLevelRunEmphasis<br>original_shape_Elongation<br>square_gldm_LargeDependenceLowGrayLevelEmphasis<br>wavelet_HLH_glszm_SmallAreaEmphasis
6mar0	0.77	0.59	5	lbp_3D_m2_glszm_GrayLevelVariance<br>log_sigma_5_0_mm_3D_glcm_Idmn<br>logarithm_firstorder_InterquartileRange<br>squareroot_glszm_LargeAreaHighGrayLevelEmphasis<br>wavelet_HLH_glszm_SizeZoneNonUniformityNormalized
6mar1	0.74	0.67	4	lbp_3D_k_firstorder_Kurtosis<br>lbp_3D_m2_firstorder_RootMeanSquared<br>log_sigma_4_0_mm_3D_glcm_Idmn<br>square_glrlm_LongRunLowGrayLevelEmphasis
6mar2	0.85	0.67	11	gradient_gldm_SmallDependenceLowGrayLevelEmphasis<br>lbp_3D_k_firstorder_Kurtosis<br>lbp_3D_m1_glszm_LowGrayLevelZoneEmphasis<br>lbp_3D_m2_firstorder_Kurtosis<br>log_sigma_3_0_mm_3D_glszm_ZoneEntropy<br>log_sigma_4_0_mm_3D_ngtdm_Contrast<br>original_gldm_LargeDependenceHigh

Supplemental materials undergo peer review but are not copyedited, and may include typographical errors.

				GrayLevelEmphasis<br>square_glcm_Correlation<br>wavelet_HHL_glcm_Imc2<br>wavelet_HLH_glszm_SizeZoneNonUniformityNormalized<br>wavelet_LHH_glszm_SmallAreaEmphasis
6mar3	0.85	0.65	9	lbp_3D_k_firstorder_Kurtosis<br>lbp_3D_m1_glszm_LowGrayLevelZoneEmphasis<br>lbp_3D_m2_firstorder_Kurtosis<br>log_sigma_5_0_mm_3D_glcm_Idmn<br>logarithm_glcm_Idmn<br>original_shape_Elongation<br>squareroot_glszm_LargeAreaHighGrayLevelEmphasis<br>wavelet_HHL_glcm_Imc2<br>wavelet_HLH_glszm_SizeZoneNonUniformityNormalized
6mar4	0.68	0.49	2	squareroot_glcm_MaximumProbability<br>wavelet_HLH_glszm_SizeZoneNonUniformityNormalized
6mar5	0.80	0.64	7	lbp_3D_k_firstorder_Kurtosis<br>lbp_3D_m2_firstorder_RootMeanSquared<br>log_sigma_4_0_mm_3D_glcm_Idmn<br>logarithm_glcm_Idmn<br>original_shape_Elongation<br>squareroot_glcm_MaximumProbability<br>wavelet_HLH_glszm_SizeZoneNonUniformityNormalized
6mar6	0.79	0.61	6	lbp_3D_k_firstorder_Kurtosis<br>lbp_3D_m2_firstorder_RootMeanSquared<br>original_shape_Elongation<br>square_glrlm_LongRunLowGrayLevelEmphasis<br>squareroot_glcm_MaximumProbabilit

Supplemental materials undergo peer review but are not copyedited, and may include typographical errors.

				y wavelet_HLH_glszm_SizeZoneNonUniformityNormalized
6mar7	0.76	0.56	4	gradient_gldm_SmallDependenceLowGrayLevelEmphasis square_glrlm_LongRunLowGrayLevelEmphasis squareroot_glcm_MaximumProbability wavelet_HLH_glszm_SizeZoneNonUniformityNormalized
6mar8	0.77	0.59	5	lbp_3D_k_firstorder_Kurtosis original_shape_Elongation square_glrlm_LongRunLowGrayLevelEmphasis squareroot_glcm_MaximumProbability wavelet_HLH_glszm_SizeZoneNonUniformityNormalized
6mar9	0.77	0.59	5	lbp_3D_k_firstorder_Kurtosis original_shape_Elongation square_glrlm_LongRunLowGrayLevelEmphasis squareroot_glcm_MaximumProbability wavelet_HLH_glszm_SizeZoneNonUniformityNormalized
6mar10	0.77	0.60	5	lbp_3D_k_firstorder_Kurtosis original_shape_Elongation square_glrlm_LongRunLowGrayLevelEmphasis squareroot_glcm_MaximumProbability wavelet_HLH_glszm_SizeZoneNonUniformityNormalized
7mar0	0.76	0.63	5	exponential_ngtdm_Busyness lbp_3D_m1_glszm_GrayLevelNonUniformityNormalized lbp_3D_m2_glcm_Imc1

Supplemental materials undergo peer review but are not copyedited, and may include typographical errors.

				log_sigma_5_0_mm_3D_glcm_Idn<br>logarithm_ngtdm_Strength
7mar1	0.80	0.63	7	lbp_3D_k_firstorder_Kurtosis<br>lbp_3D_m1_glszm_LowGrayLevelZoneEmphasis<br>lbp_3D_m2_firstorder_Kurtosis<br>log_sigma_5_0_mm_3D_glcm_Idn<br>logarithm_ngtdm_Strength<br>original_shape_Elongation<br>squareroot_glszm_LargeAreaHighGrayLevelEmphasis
7mar2	0.79	0.60	6	lbp_3D_m1_glszm_GrayLevelNonUniformityNormalized<br>lbp_3D_m2_glcm_Imc1<br>log_sigma_5_0_mm_3D_glcm_Idn<br>logarithm_ngtdm_Strength<br>original_shape_Elongation<br>squareroot_glszm_LargeAreaHighGrayLevelEmphasis
7mar3	0.72	0.57	3	lbp_3D_m2_firstorder_RootMeanSquared<br>log_sigma_5_0_mm_3D_glcm_Idmn<br>squareroot_glszm_LargeAreaHighGrayLevelEmphasis
7mar4	0.62	0.58	1	lbp_3D_m2_glcm_Imc1
7mar5	0.69	0.55	2	gradient_gldm_SmallDependenceLowGrayLevelEmphasis<br>squareroot_glcm_MaximumProbability
7mar6	0.63	0.59	1	lbp_3D_m2_glcm_Imc1
7mar7	0.67	0.61	2	lbp_3D_m2_glcm_Imc1<br>square_glszm_LargeAreaLowGrayLevelEmphasis
7mar8	0.68	0.61	2	lbp_3D_m2_glcm_Imc1<br>square_glszm_LargeAreaLowGrayLevelEmphasis

Supplemental materials undergo peer review but are not copyedited, and may include typographical errors.

7mar9	0.68	0.61	2	lbp_3D_m2_glcm_Imc1<br>square_glszm_LargeAreaLowGrayLevelEmphasis
7mar10	0.68	0.61	2	lbp_3D_m2_glcm_Imc1<br>square_glszm_LargeAreaLowGrayLevelEmphasis
8mar0	0.84	0.63	12	exponential_ngtdm_Busyness<br>lbp_3D_m1_glszm_GrayLevelNonUniformityNormalized<br>lbp_3D_m2_firstorder_90Percentile<br>lbp_3D_m2_glcm_Imc1<br>log_sigma_5_0_mm_3D_glcm_MCC<br>logarithm_ngtdm_Strength<br>original_shape_Elongation<br>squareroot_glszm_LargeAreaHighGrayLevelEmphasis<br>wavelet_HHH_glszm_LowGrayLevelZoneEmphasis<br>wavelet_HHL_glcm_Imc2<br>wavelet_HLH_glszm_SizeZoneNonUniformityNormalized<br>wavelet_LHH_glrlm_LongRunEmphasis
8mar1	0.81	0.62	9	exponential_ngtdm_Busyness<br>lbp_3D_k_firstorder_Kurtosis<br>lbp_3D_m1_glszm_GrayLevelNonUniformityNormalized<br>lbp_3D_m2_firstorder_90Percentile<br>lbp_3D_m2_glcm_Imc1<br>log_sigma_3_0_mm_3D_glcm_ClusterShade<br>logarithm_ngtdm_Strength<br>original_shape_Elongation<br>wavelet_LHH_glrlm_LongRunEmphasis
8mar2	0.78	0.59	6	lbp_3D_k_firstorder_Kurtosis<br>lbp_3D_m1_glszm_LowGrayLevelZoneEmphasis<br>logarithm_ngtdm_Strength<br>original_shape_Elongation

Supplemental materials undergo peer review but are not copyedited, and may include typographical errors.

				squareroot_glszm_LargeAreaHighGrayLevelEmphasis<br>wavelet_HLH_glszm_ZoneVariance
8mar3	0.73	0.57	5	logarithm_ngtdm_Strength<br>original_shape_Elongation<br>squareroot_glszm_LargeAreaHighGrayLevelEmphasis<br>wavelet_HLH_glszm_ZoneVariance<br>wavelet_LLL_firstorder_Skewness
8mar4	0.77	0.61	6	lbp_3D_m2_glcm_Imc1<br>logarithm_ngtdm_Strength<br>original_shape_Elongation<br>squareroot_glszm_LargeAreaHighGrayLevelEmphasis<br>wavelet_HLH_glszm_ZoneVariance<br>wavelet_LLL_firstorder_Skewness
8mar5	0.74	0.58	5	logarithm_ngtdm_Strength<br>original_shape_Elongation<br>squareroot_glszm_LargeAreaHighGrayLevelEmphasis<br>wavelet_HHH_glszm_LowGrayLevelZoneEmphasis<br>wavelet_HLH_glszm_ZoneVariance
8mar6	0.75	0.56	5	lbp_3D_m2_glcm_Imc1<br>logarithm_ngtdm_Strength<br>original_shape_Elongation<br>squareroot_glszm_LargeAreaHighGrayLevelEmphasis<br>wavelet_LLL_firstorder_Skewness
8mar7	0.77	0.61	6	lbp_3D_m2_glcm_Imc1<br>logarithm_ngtdm_Strength<br>original_shape_Elongation<br>squareroot_glszm_LargeAreaHighGrayLevelEmphasis<br>wavelet_HLH_glszm_ZoneVariance<br>wavelet_LLL_firstorder_Skewness
8mar8	0.77	0.61	5	lbp_3D_m2_glcm_Imc1<br>logarithm_ngtdm_Strength

Supplemental materials undergo peer review but are not copyedited, and may include typographical errors.

				original_shape_Elongation<br>squareroot_glszm_LargeAreaHighGrayLevelEmphasis<br>wavelet_HLH_glszm_ZoneVariance
8mar9	0.73	0.62	4	exponential_ngtdm_Busyness<br>lbp_3D_m2_glcm_Imc1<br>log_sigma_5_0_mm_3D_glcm_Idn<br>logarithm_ngtdm_Strength
8mar10	0.71	0.67	3	lbp_3D_m2_glcm_Imc1<br>log_sigma_5_0_mm_3D_glcm_Idn<br>logarithm_ngtdm_Strength
9mar0	0.83	0.69	12	exponential_ngtdm_Busyness<br>lbp_2D_glrlm_GrayLevelNonUniformityNormalized<br>lbp_3D_k_firstorder_Skewness<br>lbp_3D_m1_glszm_GrayLevelNonUniformityNormalized<br>lbp_3D_m2_firstorder_90Percentile<br>lbp_3D_m2_glcm_Imc1<br>log_sigma_3_0_mm_3D_glcm_ClusterProminence<br>log_sigma_5_0_mm_3D_glcm_InverseVariance<br>logarithm_ngtdm_Strength<br>original_shape_Elongation<br>wavelet_HHH_firstorder_Skewness<br>wavelet_HLH_glszm_LargeAreaEmphasis
9mar1	0.81	0.60	7	lbp_3D_k_firstorder_Kurtosis<br>lbp_3D_m2_glcm_Imc1<br>log_sigma_3_0_mm_3D_glcm_ClusterShade<br>logarithm_ngtdm_Strength<br>original_shape_Elongation<br>squareroot_glszm_LargeAreaHighGrayLevelEmphasis<br>wavelet_LHH_glrlm_LongRunEmphasis
9mar2	0.78	0.72	7	lbp_3D_k_firstorder_Kurtosis<br>lbp_3D_m1_glszm_GrayLevelNonUn

Supplemental materials undergo peer review but are not copyedited, and may include typographical errors.

				iformityNormalized<br>lbp_3D_m2_glcm_Imc1<br>logarithm_ngtdm_Strength<br>original_shape_Elongation<br>wavelet_HHH_firstorder_Skewness<br>wavelet_HLH_glszm_ZoneVariance
9mar3	0.77	0.69	7	lbp_3D_k_firstorder_Kurtosis<br>lbp_3D_m2_glcm_Imc1<br>log_sigma_3_0_mm_3D_glcm_ClusterProminence<br>logarithm_ngtdm_Strength<br>original_shape_Elongation<br>wavelet_HHH_firstorder_Skewness<br>wavelet_LHH_glrlm_LongRunEmphasis
9mar4	0.81	0.59	7	lbp_3D_m2_glcm_Imc1<br>log_sigma_3_0_mm_3D_glcm_ClusterProminence<br>logarithm_ngtdm_Strength<br>original_shape_Elongation<br>squareroot_glszm_LargeAreaHighGrayLevelEmphasis<br>wavelet_HHH_firstorder_Skewness<br>wavelet_LHH_glrlm_LongRunEmphasis
9mar5	0.83	0.63	9	lbp_3D_k_firstorder_Kurtosis<br>lbp_3D_m2_glcm_Imc1<br>log_sigma_3_0_mm_3D_glcm_ClusterProminence<br>logarithm_ngtdm_Strength<br>original_shape_Elongation<br>squareroot_glszm_LargeAreaHighGrayLevelEmphasis<br>wavelet_HHH_firstorder_Skewness<br>wavelet_HLH_glszm_LargeAreaEmphasis<br>wavelet_LHH_glrlm_LongRunEmphasis
9mar6	0.82	0.63	8	lbp_3D_m2_glcm_Imc1<br>log_sigma_3_0_mm_3D_glcm_Cluste

Supplemental materials undergo peer review but are not copyedited, and may include typographical errors.

				rProminence<br>logarithm_ngtdm_Strength<br>original_shape_Elongation<br>squareroot_glszm_LargeAreaHighGrayLevelEmphasis<br>wavelet_HHH_firstorder_Skewness<br>wavelet_HLH_glszm_ZoneVariance<br>wavelet_LHH_glrlm_LongRunEmphasis
9mar7	0.80	0.62	7	lbp_3D_k_firstorder_Kurtosis<br>lbp_3D_m2_glcm_Imc1<br>logarithm_ngtdm_Strength<br>original_shape_Elongation<br>squareroot_glszm_LargeAreaHighGrayLevelEmphasis<br>wavelet_HHH_firstorder_Skewness<br>wavelet_LHH_glrlm_LongRunEmphasis
9mar8	0.79	0.63	6	lbp_3D_m2_glcm_Imc1<br>logarithm_ngtdm_Strength<br>original_shape_Elongation<br>squareroot_glszm_LargeAreaHighGrayLevelEmphasis<br>wavelet_HHH_firstorder_Skewness<br>wavelet_LHH_glrlm_LongRunEmphasis
9mar9	0.81	0.64	7	lbp_3D_m2_glcm_Imc1<br>logarithm_ngtdm_Strength<br>original_shape_Elongation<br>squareroot_glszm_LargeAreaHighGrayLevelEmphasis<br>wavelet_HHH_firstorder_Skewness<br>wavelet_LHH_glrlm_LongRunEmphasis<br>wavelet_LLL_firstorder_Skewness
9mar10	0.81	0.65	7	lbp_3D_m2_glcm_Imc1<br>logarithm_ngtdm_Strength<br>original_shape_Elongation<br>squareroot_glszm_LargeAreaHighGrayLevelEmphasis

Supplemental materials undergo peer review but are not copyedited, and may include typographical errors.

				wavelet_HHH_firstorder_Skewness<br>wavelet_LHH_glrlm_LongRunEmphasis<br>wavelet_LLL_firstorder_Skewness
10mar0	0.81	0.68	10	exponential_ngtdm_Busyness<br>lbp_3D_k_firstorder_Kurtosis<br>lbp_3D_m2_firstorder_90Percentile<br>lbp_3D_m2_glcm_MCC<br>log_sigma_5_0_mm_3D_glcm_InverseVariance<br>logarithm_ngtdm_Strength<br>original_shape_Elongation<br>wavelet_HHH_firstorder_Skewness<br>wavelet_HLH_glszm_SizeZoneNonUniformityNormalized<br>wavelet_LHH_glrlm_LongRunEmphasis
10mar1	0.82	0.68	7	exponential_ngtdm_Busyness<br>lbp_3D_k_firstorder_Kurtosis<br>lbp_3D_m2_glcm_Imc1<br>logarithm_ngtdm_Strength<br>original_shape_Elongation<br>wavelet_LHH_glrlm_LongRunEmphasis<br>wavelet_LLL_firstorder_Skewness
10mar2	0.80	0.68	7	exponential_ngtdm_Busyness<br>lbp_3D_k_firstorder_Skewness<br>lbp_3D_m2_glcm_Imc1<br>logarithm_ngtdm_Strength<br>original_shape_Elongation<br>wavelet_HHH_firstorder_Skewness<br>wavelet_LHH_glrlm_LongRunEmphasis
10mar3	0.84	0.60	9	exponential_ngtdm_Busyness<br>lbp_3D_k_firstorder_Kurtosis<br>lbp_3D_m2_glcm_Imc1<br>logarithm_ngtdm_Strength<br>original_shape_Elongation<br>squareroot_glszm_LargeAreaHighGrayLevelEmphasis

Supplemental materials undergo peer review but are not copyedited, and may include typographical errors.

				wavelet_HHH_glszm_ZoneEntropy wavelet_HLH_glszm_SizeZoneNonUniformityNormalized wavelet_LHH_glrlm_LongRunEmphasis
10mar4	0.78	0.57	6	lbp_3D_k_firstorder_Kurtosis lbp_3D_m2_glcm_Imc1 logarithm_ngtdm_Strength original_shape_Elongation squareroot_glszm_LargeAreaHighGrayLevelEmphasis wavelet_HHH_firstorder_Skewness
10mar5	0.81	0.61	7	lbp_3D_k_firstorder_Kurtosis lbp_3D_m2_glcm_Imc1 logarithm_ngtdm_Strength original_shape_Elongation squareroot_glszm_LargeAreaHighGrayLevelEmphasis wavelet_HHH_firstorder_Skewness wavelet_LHH_glrlm_LongRunEmphasis
10mar6	0.80	0.62	6	lbp_3D_m2_glcm_Imc1 logarithm_ngtdm_Strength original_shape_Elongation squareroot_glszm_LargeAreaHighGrayLevelEmphasis wavelet_HHH_firstorder_Skewness wavelet_LHH_glrlm_LongRunEmphasis
10mar7	0.81	0.62	7	lbp_3D_k_firstorder_Skewness lbp_3D_m2_glcm_Imc1 logarithm_ngtdm_Strength original_shape_Elongation squareroot_glszm_LargeAreaHighGrayLevelEmphasis wavelet_HHH_firstorder_Skewness wavelet_LHH_glrlm_LongRunEmphasis
10mar8	0.78	0.59	6	lbp_3D_k_firstorder_Kurtosis

Supplemental materials undergo peer review but are not copyedited, and may include typographical errors.

				lbp_3D_m2_glcm_Imc1<br>logarithm_ngtdm_Strength<br>original_shape_Elongation<br>squareroot_glszm_LargeAreaHighGra<br>yLevelEmphasis<br>wavelet_HHH_firstorder_Skewness
10mar9	0.77	0.59	5	lbp_3D_m2_glcm_Imc1<br>logarithm_ngtdm_Strength<br>original_shape_Elongation<br>squareroot_glszm_LargeAreaHighGra<br>yLevelEmphasis<br>wavelet_HHH_firstorder_Skewness
10mar10	0.81	0.63	7	lbp_3D_m2_glcm_Imc1<br>logarithm_ngtdm_Strength<br>original_gldm_LargeDependenceHigh<br>GrayLevelEmphasis<br>original_shape_Elongation<br>squareroot_glszm_ZoneVariance<br>wavelet_HHH_firstorder_Skewness<br>wavelet_HLH_glszm_ZoneVariance
Orginal	0.71	0.59	2	lbp_3D_m2_glszm_HighGrayLevelZo<br>neEmphasis<br>wavelet_HLL_glcm_MCC

AUC: Area Under the Receiver Operating Characteristic Curve; Original: Refers to the original lesion region.

Table S5: Reproducibility of the Selected Radiomic Features (Intra-class Correlation Coefficient Analysis)

**Features**	**ICC**	**CI lower**	**CI upper**	***P value***
logarithm_firstorder_Minimum	0.7984	0.6188	0.8987	<.001
logarithm_glcm_Imc1	0.9152	0.8297	0.9587	<.001
original_shape_Elongation	0.85	0.7089	0.9257	<.001
wavelet_LHL_glcm_MCC	0.8472	0.704	0.9243	<.001
wavelet_HLH_ngtdm_Busyness	0.7756	0.5804	0.8865	<.001
log_sigma_3_0_mm_3D_glcm_ClusterShade	0.808	0.6351	0.9038	<.001
wavelet_HHH_glcm_JointEntropy	0.9078	0.8157	0.955	<.001

P value: Calculated using a two-way random-effects model, the *P value* tests the null hypothesis

Supplemental materials undergo peer review but are not copyedited, and may include typographical errors.

that the ICC equals zero. ICC: Intraclass Correlation Coefficient; CI: Confidence Interval.

Table S6: Diagnostic Performance of the Radiomics-Based OMR Model at Different Rad-Score Cutoff Values in the Training Cohort.

Cutoff	Sensitivity	Specificity	PPV	NPV	Accuracy	Youden Index
0.25	**200/254 (0.789)**	167/254 (0.657)	126/254 (0.496)	223/254 (0.880)	177/254 (0.697)	0.447
0.30	181/254 (0.711)	194/254 (0.764)	143/254 (0.562)	219/254 (0.861)	190/254 (0.748)	0.475
0.35	167/254 (0.658)	207/254 (0.815)	153/254 (0.602)	215/254 (0.848)	195/254 (0.768)	0.473
0.40	147/254 (0.579)	217/254 (0.854)	160/254 (0.629)	210/254 (0.826)	196/254 (0.772)	0.433
0.45	120/254 (0.474)	228/254 (0.899)	170/254 (0.667)	203/254 (0.800)	196/254 (0.772)	0.373
0.50	104/254 (0.408)	**234/254 (0.921)**	**175/254 (0.689)**	199/254 (0.785)	195/254 (0.768)	0.329
Mean: 0.299	167/254 (0.658)	207/254 (0.815)	153/254 (0.602)	215/254 (0.848)	195/254 (0.768)	0.433
Median:0.238	162/254 (0.637)	197/254 (0.775)	148/254 (0.582)	213/254 (0.841)	187/254 (0.734)	0.413
Our: 0.334	181/254 (0.711)	207/254 (0.815)	158/254 (0.621)	**220/254 (0.868)**	**199/254 (0.783)**	**0.525**

Bolded values in the table indicate the optimal performance metrics. PPV: Positive Predictive Value; NPV: Negative Predictive Value.

Table S7: Tumor margin radiological features: Definitions and Descriptions

**Characteristic**	Definition
Peripheral "washout"	Phenomenon observed in liver imaging where lesions, typically located in the peripheral regions of the liver, exhibit a gradual decrease in enhancement relative to the surrounding composite liver tissue from earlier to later phases. This process results in hypoenhancement during the extracellular phase.
Capsule integrity	During the portal venous or delayed phase, the lesion exhibits a sharp, well-defined enhancement at the periphery. The degree of enhancement is higher than that of the tumor and adjacent liver parenchyma. (No: enhancement appear incomplete;Yes: enhancement

Supplemental materials undergo peer review but are not copyedited, and may include typographical errors.

	appear complete;None:no enhancement)
Halo like enhancement	Lesions show ring-like or halo-like enhancement areas around them.
High EOB uptake	The marginal area of liver cancer may show high uptake of liver-specific MRI contrast agent (Gd-EOB-DTPA).
Peritumoral low signal	Low signal area around the tumor.
Tumor morphology (38)	N: nodular — nodules manifest as distinct, typically circumscribed lesions; CM: nodular with extranodular growth — extranodular growth manifests as a focal extension of the tumor beyond the expected margin of a nodule,irregular margins of a nodule, or satellite nodules; NEG :confluent multinodular—synchronous or metachronous nodules, which, due to proximity and growth, have become confluent and resemble a single mass.

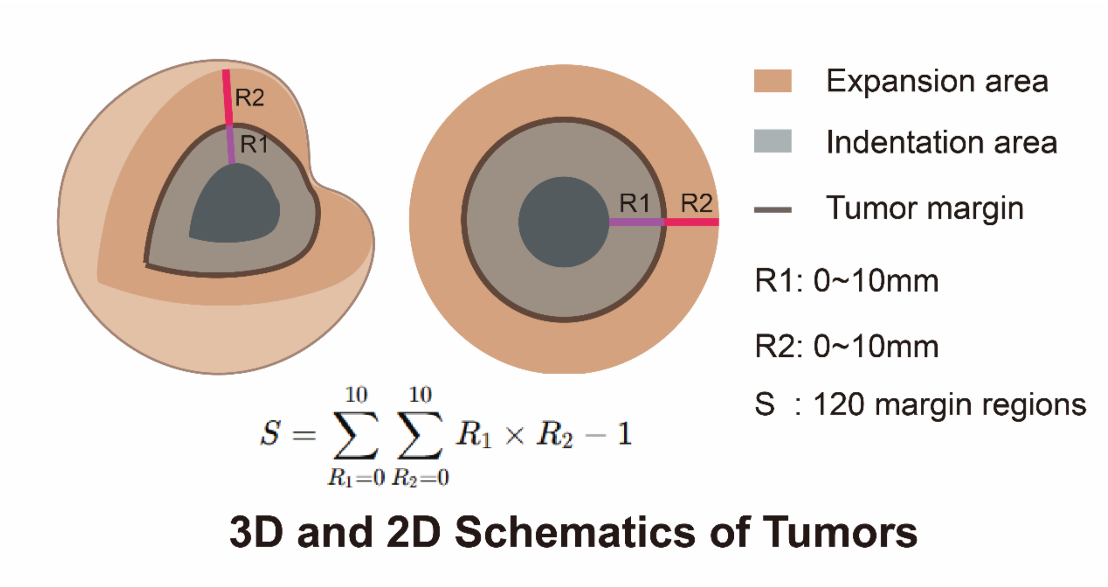

Figure S1: Definition of tumor VOI areas.

Supplemental materials undergo peer review but are not copyedited, and may include typographical errors.

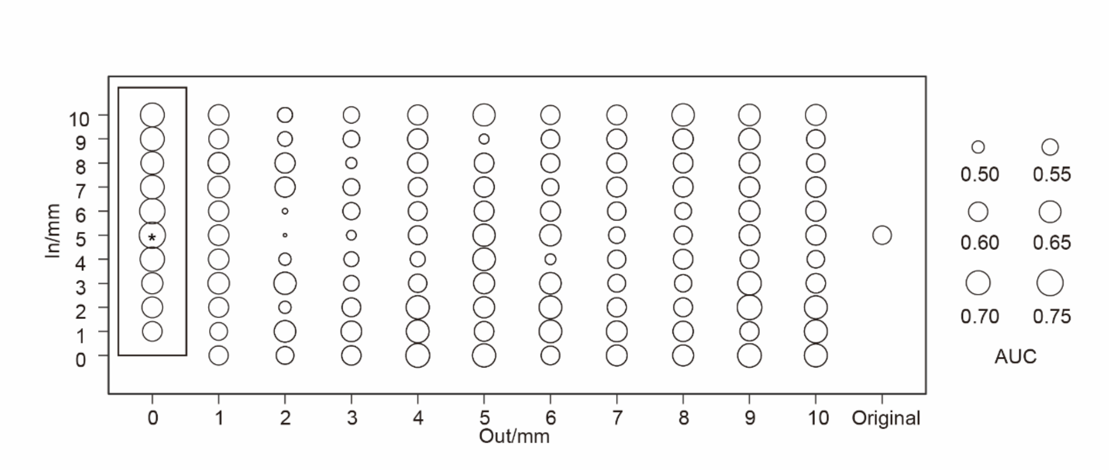

Figure S2: Display of AUC sizes of OMR regions across 121 VOIs. Y-axis: inward (In) margin shrinkage distance (0–10 mm).X-axis: outward (Out) margin expansion distance (0–10 mm). Original: the unmodified tumor without margin adjustment. Asterisk (*): the optimal margin region (OMR) with the highest AUC. Box: Stable and high AUCs across different inner margins.

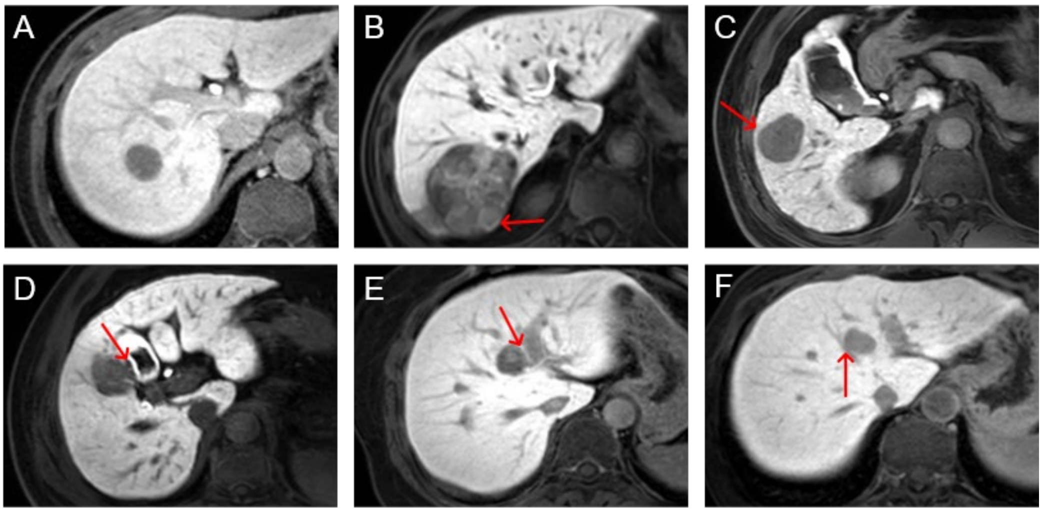

Figure S3: Representative images showing VOI expansion and involvement of adjacent structures. (A) VOI expansion without contacting any surrounding tissues. (B) Lesion itself extending beyond the liver boundary. (C) VOI exceeding the liver boundary after expansion. (D) VOI involving the gallbladder after expansion. (E) VOI involving the portal vein after expansion. (F) VOI involving small vascular structures after expansion.

Supplemental materials undergo peer review but are not copyedited, and may include typographical errors.

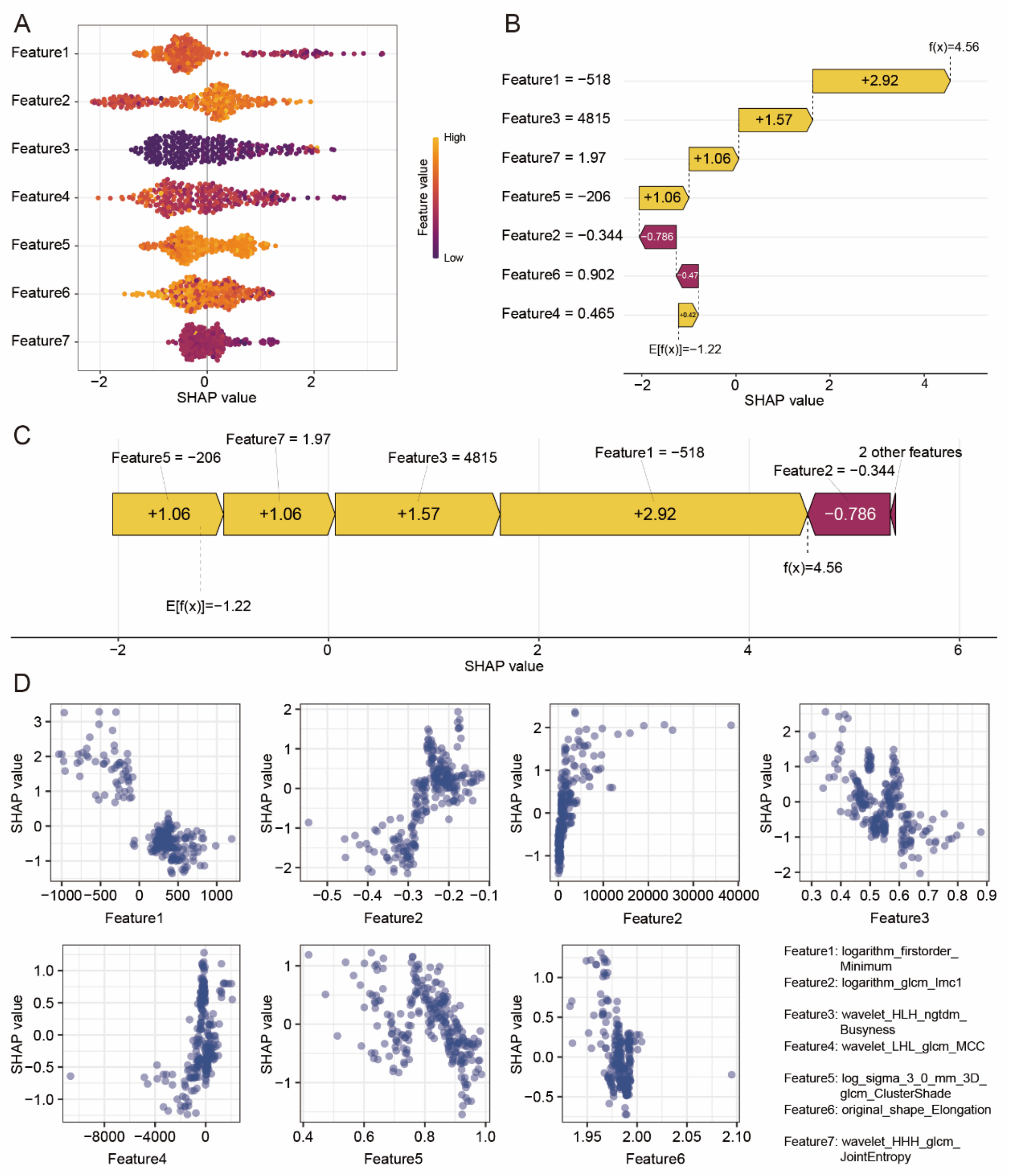

Figure S4: SHAP plots illustrating feature impacts on model prediction. (A) SHAP value distribution for features; x-axis: SHAP values, y-axis: features, color: feature values. (B) Single-sample SHAP breakdown showing positive (yellow) and negative (purple) contributions from baseline to prediction. (C) Simplified single-sample plot highlighting main features, merging minor ones. (D) Scatter plots of feature values vs. SHAP values showing their relationship.

Supplemental materials undergo peer review but are not copyedited, and may include typographical errors.

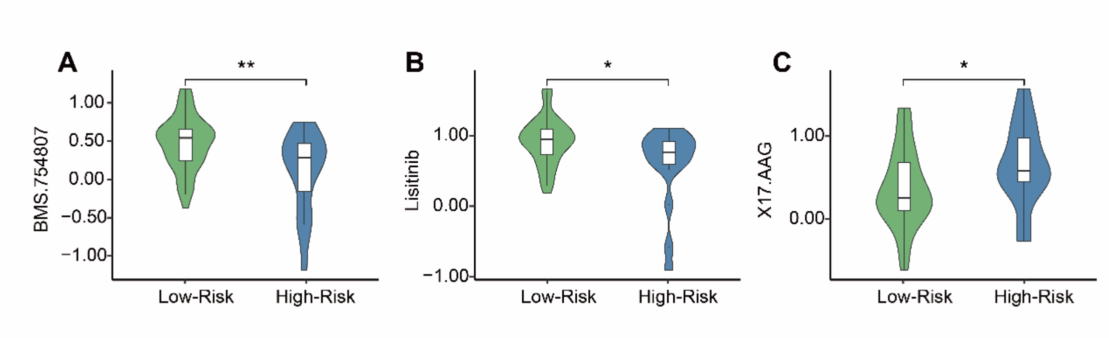

Figure S5: (A-C) Sensitivity analysis violin plot. IC50 analysis of differential gene-drug sensitivity in two groups stratified using the OMR model based on differential genes identified in the RNA-seq cohort.
